# The Genus *Eriosema* (Fabaceae): From the Ethnopharmacology to an Evidence-Based Phytotherapeutic Perspective?

**DOI:** 10.3389/fphar.2021.641225

**Published:** 2021-05-07

**Authors:** Sylvin Benjamin Ateba, Dieudonné Njamen, Liselotte Krenn

**Affiliations:** ^1^ Department of Biology of Animal Organisms, Faculty of Science, University of Douala, Douala, Cameroon; ^2^ Laboratory of Animal Physiology, Department of Animal Biology and Physiology, Faculty of Science, University of Yaoundé I, Yaoundé, Cameroon; ^3^ Department of Pharmacognosy, University of Vienna, Vienna, Austria

**Keywords:** *Eriosema*, Fabaceae, pharmacological activities, phytochemistry, ethnopharmacology, toxicology

## Abstract

The genus *Eriosema* (Fabaceae) includes approximately 150 species widely distributed across tropical and subtropical regions of the world (Africa, Neotropics, Asia and Australia). Throughout these regions, several species are used since centuries in different traditional medicinal systems, while others are used as food or food supplement. The present review attempts to critically summarize current information concerning the uses, phytochemistry and pharmacology of the *Eriosema* genus and to evaluate the therapeutic potential. The information published in English and French (up to September 2020) on ethnopharmacology or traditional uses, chemistry, pharmacology and toxicology of *Eriosema* genus was collected from electronic databases [SciFinder, PubMed, Google, Google Scholar, Scopus, Web of Science, Prelude Medicinal Plants—http://www.ethnopharmacologia.org/recherche-dans-prelude/?plant, The Plant List (http://www.theplantlist.org/), POWO (http://powo.science.kew.org/) and IUCN Red List Categories (https://www.iucnredlist.org/)], conference proceedings, books, M.Sc. and Ph.D. dissertations. The information retrieved on the ethnomedicinal indications of *Eriosema* genus allowed to list 25 species (∼16.6% of the genus). The majority of uses is recorded from Africa. Phytochemical analyses of 8 species led to the identification and/or isolation of 107 compounds, with flavonoids (69.2%), chromones (7.5%) and benzoic acid derivatives (3.7%) as the main chemical classes. Pharmacological investigations with crude extracts and isolated compounds showed a broad range of activities including aphrodisiac, estrogenic, anti-osteoporosis, hypolipidemic, anti-diabetic, anti-diarrheal, anti-microbial, anti-oxidant, anthelmintic, anti-cancer, and acetylcholinesterase inhibitory activities. Despite the low number of *Eriosema* species tested, there is convincing evidence in *vitro* and *in vivo* studies validating some traditional and ethnobotanical uses. However, the utility of several of the described uses has not yet been confirmed in pharmacological studies. Reviewed data could serve as a reference tool and preliminary information for advanced research on *Eriosema* species.

## Introduction

*Eriosema* (Fabaceae), with approximately 150 species of shrubs, shrublets and herbs, is the second-largest genus of the Cajaninae subtribe (Ma et al., 1995; LPWG, 2017). This monophyletic clade originated in the late Miocene and its diversification occurred in parallel with the savanna biome expansion around the world (Cândido et al., 2020). The current centers of diversity and endemism of *Eriosema* species include the grasslands, wooded savanna and waste places of Africa (West, Central, East and South Africa), Central America (from Mexico to northern Argentina, except for Chile), Southeast Asia and Northern Australia (Wu, 1991; Schrire et al., 2005; Cândido et al., 2019). The African species are most abundant (nearly 110 species) and the first diverging evolution suggests an African origin of the genus (Cândido et al., 2020). The study by Cândido et al. (2019) recorded 35 *Eriosema* species in Brazil, which encompasses 85% of the diversity of the genus in the Americas (35 out of 41 species).

Throughout this pantropical region, several *Eriosema* species are intensively used in traditional medicine and for non-medicinal purposes (e.g. food/vegetables, tooth brush). Based on the traditional uses, phytochemical, pharmacological and toxicological investigations of different *Eriosema* species have been performed. Nevertheless, till now only few reviews dealing with *Eriosema* species have been published. The studies by Cândido et al. (2019, 2020), respectively, focused on the taxonomic synopsis of *Eriosema* in Brazil and molecular phylogenetic insights into the evolution of *Eriosema*. In 2015, Awouafack et al. demonstrated that *Eriosema* species represent a rich source of flavonoids with interesting pharmacological activities. In this review, authors compiled a total of 52 flavonoids (isoflavones, dihydroflavanols, isoflavanones, flavanols, flavanones, and dihydrochalcones) isolated from *Eriosema* species. However, the pharmacological properties are not limited to the flavonoids. In the literature, compounds from other chemical classes as well as plant extracts (more easily available and affordable than isolated compounds) demonstrated pharmacological properties. More recently, the review of Kleynhans et al. (2020) focused on *Eriosema kraussianum* Meisn. None of these reviews presented the global overview on the traditional uses, phytochemistry, pharmacological properties and toxicological evaluation of this genus. Accordingly, the present review attempts to critically summarize the available information on the traditional uses, phytochemistry, and pharmacological activities as well as safety evaluation of the genus *Eriosema* and, therefore to evaluate the data for potential phytotherapeutic approaches.

## Search Strategy and Terms Used

The information on the genus *Eriosema* published up to September 2020 in English and French was collected from different electronic data bases (SciFinder including Chemical Abstracts and Medline, PubMed, Google, Google Scholar, Scopus, Web of Science), conference proceedings, books, M.Sc. and Ph.D. dissertations. The keywords “*Eriosema*” in conjunction with ‘traditional uses’ or ‘ethnopharmacology,’ ‘phytochemistry,’ ‘pharmacology,’ and ‘toxicology’ were used. In addition, Prelude Medicinal Plants (http://www.ethnopharmacologia.org/recherche-dans-prelude/?plant), The Plant List (http://www.theplantlist.org/) and POWO (http://powo.science.kew.org/) were explored for the plant´s traditional uses and scientific names, while information on the global extinction risk status of *Eriosema* species was gathered from the IUCN (International Union for Conservation of Nature) Red List Categories (https://www.iucnredlist.org/).

## Traditional Uses and Ethnopharmacology of *Eriosema* Species

Since centuries, *Eriosema* species have widely been traditionally used as household remedies against various human ailments. Accordingly, in the pharmaceutical landscape this genus may represent a remarkable source of promising substances. From the literature review, various medicinal and contemporary uses associated with this genus are summarized in Table 1.

**TABLE 1:** Ethnomedicinal indications and local uses of *Eriosema* species based on the literature data review.

Species	Parts used	Location	Local and traditional uses	Preparation	References
*Eriosema affine* De Wild.	Roots	Angola (Bié province)	Malaria, stomach pain, diarrhea	Decoction	Novotna et al. (2020)
		Angola	Prevention of abortion, chest pain, malaria, vomiting, nightmares, epilepsy, flu	Not specified	Bossard (1996)
*Eriosema benthamianum* Mart. ex Benth.	Roots	Brazil	Inflammation	Not specified	Hirschhorn (1982)
*Eriosema burkei* Benth. ex Harv. & Sond.	Roots	Malawi	Pain, lymphoid disorders	Water extract	Msonthi and Magombo (1983)
*Eriosema campestre* var. *macrophyllum* (Gear) Fortunato.	Roots	Brazil (Brazilian Cerrado)	Inflammatory diseases e.g. inflammatory skin disorders such as psoriasis	Decoction	Santos et al. (2016)
*Eriosema chinense* Vogel (syn. *Eriosema himalaicum* H.Ohashi or *Eriosema tuberosum* (Ham.) Wang et Tang)	Roots	Northern Australia, China, North East of India	Food/vegetable, diarrhea	Consumption of fresh roots	Neogi et al. (1989); Martin and Ryan (2004); Prasad et al. (2013c)
		China	Diarrhea, orchitis, detoxification	Not specified	Kunming Institute of Botany (1979)
		Thailand	Tonic	Consumption of fresh roots	Thongnest et al. (2013)
	Seeds, leaves	India (Meghalaya)	Diarrhea, wounds, astringent, diuretic, tonic, cold sweats, and parturition (promoting discharge of lochia)	Decoction	Prasad et al. (2013c), Ashraf and Borthakur (2005), Laloo and Hemalatha (2011), Thongnest et al. (2013)
	Seeds	Thailand (central region of Myanmar)	Scrofula, diarrhea, leucorrhoea, menstrual problems	Decoction	Aye et al. (2019)
			Wound healing and diuretic	Decoction	Thongnest et al. (2013)
*Eriosema cordatum* E.Mey	Roots	South Africa	Male infertility, erectile dysfunction, impotence	Hot milk infusion; decoction	Drewes et al. (2002), Drewes et al. (2013)
			Aphrodisiac mixed with *Corchorus asplenifolius* Burch.		Hutchings et al. (1996)
			Female infertility, impotence	Not specified	Bryant (1966)
	Leaves	Republic of Congo	Eye diseases	Eye instillation of fresh leavesʼ juice	Kimpouni et al. (2018)
*Eriosema crinitum* (Kunth) G. Don	Roots	Brazilia (Cerrado savannahs)	Abortive, contraceptive	Decoction	Rodrigues (2007)
*Eriosema diffusum* (Kunth) G. Don	Whole plant	Mexico Guatemala	Unspecified female diseases	Not specified	Hastings (1990)
*Eriosema englerianum* Harms	Roots	Zimbabwe	Bilharziosis	Not specified	Mmbengwa et al. (2009)
*Eriosema ellipticifolium* Schinz	Fruit	South Africa	Dietary supplement	Consumption of fruits	Mabogo (1990)
*Eriosema glomeratum* (Guill. & Perr.) Hook.f	Leaves (petiole)	Gabon (Ngounie province)	Syncope, fish-poison	Decoction	Akendengué and Louis (1994)
		Benin (Sudano-Guinean zone)	Diabetes	Maceration	Lawin et al. (2015)
	Leaves	Angola (Bakongo tribes)	Diarrhea, dysentery, cholera, shigellosis	Consumption of fresh leaves	Göhre et al. (2016)
		Angola (province of Uíge)	Food/vegetables	Consumption of fresh leaves, tea	Lautenschläger et al. (2018)
			Vertigo fainting, syncope	Maceration (bath)	
	Leafy stem	Benin	Delayed closure of fontanel	Decoction	Adomou et al. (2012)
	Roots	Burkina Faso	Hernia	Dried, pounded roots in coffee or porridge	Ouoba et al. (2006)
	Whole plant	Cameroon (Bakola Pygmy tribe)	Pulmonary troubles, mucosal infections, nasopharyngeal infections, leprosy, skin infections, venereal diseases	Decoction	Verdcourt (1970)
*Eriosema griseum* Baker	Leaves	Ivory Coast	Parasitic diseases associated with stomach ache, dysentery, diarrhea in children	Not specified	Koné et al. (2005), Koné et al. (2012)
*Eriosema kraussianum* Meisn.	Roots	South Africa	Erectile dysfunction, impotence, urinary complaints in males	Hot milk infusion of roots; maceration of root bark	Drewes et al. (2002), Drewes et al. (2004)
					Bryant (1966); Hutchings et al. (1996)
*Eriosema laurentii* De Wild.	Leaves	West Africa	Food/vegetable, nervous disorders, laxative, leprosy, nasopharyngeal affections, pulmonary problems, skin diseases, mucosal infections, venereal diseases, fish-poison	Not specified	Burkill (1985)
	Leaves, roots	Cameroon	Infertility, gynecological and menopausal complaints	Decoction	Ateba et al. (2013b)
*Eriosema lebrunii* Staner & De Craene	Leaves	Burundi	Skin diseases, eczema, impetigo, dermatoses, ringworm, fungal infections, athlete's foot, urticaria	Application of juice of fresh leaves on the infection site	Ngezahayo et al. (2015)
*Eriosema montanum* Bak. f.	Not specified	Democratic Republic of Congo	Wound healing, antimicrobial, disinfectant	Not specified	Kasonia et al. (1991)
	Leaves	Burundi	Injuries	Application of pounded fresh or dry leaves on the injury	Byavu et al. (2000)
			Oxytocic during childbirth	Decoction	Lewalle and Rodegem (1968)
			Impotence, anemia, vitamin deficiency, delayed motor development, virility, metrorrhagia, overwork, tonic	Decoction, maceration, enema	Polygenis-Bigendako (1990); Baerts and Lehmann (1989)
			Otitis, mumps, deafness, earache, otorrhea	Ear instillation of fresh leaves´ juice	Polygenis-Bigendako (1990); Van Puyvelde et al. (1977)
			Snake bite, sting of poisonous animals	Application of pounded leaves at the bite site, decoction	Polygenis-Bigendako (1990); Fumba (1983); Van Puyvelde et al. (1977); Baerts and Lehmann (1989)
			Skin diseases (ringworm, mycosis, dermatosis, eczema)	Consumption of calcined dried leaves	Ngezahayo et al. (2015)
			Diarrhea, dysentery, cholera	Decoction, enema	Ngezahayo et al. (2015); Baerts and Lehmann (1989)
			Joint pain, inflammation, rheumatism	Macerate; vigorous local rubbing of leaves	Van Puyvelde et al. (1977)
			Eye diseases	Eye instillation of fresh leaves´ juice	Baerts and Lehmann (1989)
			Sprain, hematoma, strain, dislocation, contusion, fracture	Local application of pounded fresh leaves	
			Palpitation, heart pain	Not specified	
			Pulmonary problems, cough, fever, vomiting, nausea, anxiety, epilepsy, depression, nervous disorders, mental illnesses, analgesic	Decoction, steam bath	
		Rwanda	Snake-bites, cough, conjunctivitis	Not specified	Baerts and Lehmann (1989); Rwangabo (1993)
	Roots	Rwanda	Pulmonary problems, cough	Consumption of raw roots	Durand (1960)
*Eriosema parviflorum* E.Mey	Leaves, bark, roots	Tanzania (Kagera and Lindi regions)	Malaria	Decoction, juice of fresh leaves	Nondo et al. (2015); Moshi et al. (2010)
*Eriosema psoraleoides* (Lam.) G.Don	Twigs	Tanzania (Morogoro)	Tooth brush	Not specified	Khan et al. (2000)
	Stem bark	Democratic Republic of Congo (Kinshasa)	Tuberculosis	Decoction	Ngbolua et al. (2014)
		Central African Republic	Laxative	Not specified	Sandberg (1965)
		Republic of Congo			
	Leaves	Tanzania (Bukoba rural district)	Health problems related to HIV/AIDS such as chronic diarrhea	Not specified	Kisangau et al. (2007)
		Central Africa	Oxytocic during childbirth	Water extract	Sillans (1953)
		West Africa	Ectoparasites	Rubbing locally	Dalziel (1937)
		Central African Republic	Endoparasites (cestodes, nematodes, tapeworms, amoebiasis)	Decoction	Wome (1985), Sillans (1953)
			Diarrhea, dysentery	Maceration	Haxaire (1979)
			Vaginal prolapse, wound healing	Fumigation	Haxaire (1979), Descoings (1963)
			Eye diseases	Not specified	Descoings (1963)
		Cameroon	Eye diseases	Maceration (eyewash)	Malzy (1954)
		Democratic Republic of Congo	Expectorant	Decoction	Goossens (1924)
			Fish-poison	Not specified	Goossens (1924)
			Eye diseases	Eye instillation of fresh leaves´ juice	Wome (1985)
			Wound healing	Application of pounded dried leaves on the wound	Nyakabwa and Gapusi (1990)
			Abscess, boil, ulcer, acne	Local application of pounded fresh leaves	Nyakabwa and Gapusi (1990)
			Sexually transmited diseases, expectorant	Decoction	Staner and Boutique (1937)
		Ivory Coast, Burkina Faso	Eye diseases	Eye instillation of fresh leaves´ juice	Kerharo and Bouquet (1950)
		Benin	Skin diseases	Local application of fresh leaves´ juice	Verger (1995)
		Nigeria	Skin diseases, fever, malaria	Decoction	Adjanohoun et al. (1991)
		Rwanda	Laxative, placental expulsion, pulmonary problems, cough	Decoction	Desouter (1991)
		Burundi	Tonic	Leaf juice	Baerts and Lehmann (1989)
	Roots	Tanganyika	Miscarriage in combination with *Piliostigma trionningii*	Water extract	Haerdi (1964)
		Central African Republic	Otitis, mumps, deafness, earache, otorrhea	Ear instillation of fresh leaves´ juice	Boulesteix et al. (1979)
			Wound healing	Application of scraped root on the wound	Wome (1985)
		Democratic Republic of Congo	Sexually transmited diseases	Maceration	Staner and Boutique (1937)
		Uganda	Stomach ache, gastritis, colic, colitis, stomach ulcer	Decoction	Adjanohoun (1993)
		Rwanda	Pulmonary problems	Consumption of roots	Durand (1960)
		Burundi	Sexually transmited diseases	Decoction	Baerts and Lehmann (1989)
	Roots, leaves	Kenya	Abdominal pain, gastritis, stomach aches	Decoction	Masinde (1996)
		Tanzania	Febrile convulsions, fever, malaria, impotence, frigidity, erectile dysfunction	Decoction	Moshi et al. (2009), Moshi et al. (2010)
			Urogenital infections, miscarriage, ovarian cyst, pelvic pain	Not specified	Haerdi (1964)
	Whole plant	Burundi	Diarrhea, dysentery, cholera	Decoction	Polygenis-Bigendako (1990)
	Leafy stem	Benin	Jaundice, liver failure, cirrhosis	Not specified	Adjanohoun et al. (1989)
		Togo	Dysmenorrhea, hypermenorrhea	Decoction	Adjanohoun et al. (1986)
	Not specified	Uganda (Sango Bay forest reserve)	Malaria	Not specified	Galabuzi et al. (2016)
		Republic of Congo	Endoparasites	Not specified	Bouquet (1969)
*Eriosema pulchellum* (Kunth) G.Don	Roots	Central African Republic	Infertility, impotence	Decoction	Vergiat (1969)
*Eriosema robustum* Baker	Not specified	East Africa	Coughs	Not specified	Kokwaro (2009)
		Cameroon	Skin diseases	Not specified	Awouafack et al. (2013a)
*Eriosema rufum* (Kunth) G.Don	Roots	Venezuela	Women´s sterility, parturition	Not specified	Morton (1981)
*Eriosema salignum* E.Mey	Roots, rootbark	South Africa	Male sexual disorders, erectile dysfunction, impotence	Hot milk infusion of roots, maceration of root bark	Drewes et al. (2002), Drewes et al. (2013)
					Hulme (1954)
*Eriosema scioanum* Avetta	Leaves	Ethiopia	Sexually transmited diseases	Not specified	Lemordant (1972)
*Eriosema stanerianum* Hauman	Leaves	Uganda (Sango Bay area)	Malaria	Decoction	Ssegawa and Kasenene (2007)
*Eriosema tisserantii* Staner & De Craene	Roots	Central Africa	Aphrodisiac, erectile dysfunction	Water extract	Sillans (1953)
		Central African Republic	Women’s sterility, frigidity	Decoction	Vergiat (1969)

According to South African Zulu traditional health practitioners, the roots of several *Eriosema* species such as *Eriosema kraussianum* N. E. Br., *Eriosema salignum* E. Mey. and *Eriosema cordatum* E. Mey., known under the indigenous umbrella name of *uBangalala*, are effective to cure or alleviate erectile dysfunction (ED) and/or impotence (Bryant, 1966; Hutchings et al., 1996; Ojewole et al., 2007; Drewes et al., 2013). In Southern and Eastern Africa, they are also used as expectorants and diuretics (Watt and Breyer-Brandwijk, 1962). In case of impotence, hot milk infusions of the plants´ roots and/or pounded boiled root decoctions are taken at small doses twice a day (Hulme, 1954; Bryant, 1966). Studies reported that the roots of *E. cordatum* are used for male (Hutchings et al., 1996) and female (Bryant, 1966) infertilities and as constituent of *Imbiza ephuzwato*, a Zulu traditional herbal medicine (Van Wyk et al., 1997). This mixture is used as tonic, against anxiety, for clearing skin conditions, to treat diabetes mellitus, kidney and urinary infections, tonsillitis, pneumonia, constipation, stomach and back pains, for the improvement of high blood pressure, to boost energy, vitality and sexual activity as well as in the prevention of arthritis (Ndhlala et al., 2011).

In Angola, fresh leaves of *Eriosema glomeratum* (Guill. & Perr.) Hook. f. are eaten as food/vegetables and administered against diarrhea and dysentery (Göhre et al., 2016; Lautenschläger et al., 2018). In Benin, they are used against diabetes (Lawin et al., 2015), while the leafy stem is claimed to promote the closure of fotannel (Adomou et al., 2012). The roots are applied in Burkina Faso against hernia (Ouoba et al., 2006). In the Bakola Pygmy tribe, the whole plant is used for many purposes including pulmonary troubles, nasopharyngeal infections, leprosy and venereal diseases (Verdcourt, 1970).

Indians around Kunana (Venezuela) use the root decoction of *Eriosema rufum* G. Don. against sterility in women and to accelerate delivery in childbirth (Morton, 1981).

*Eriosema robustum* Baker is widespread in Burundi, Ethiopia, Kenya, Rwanda, Tanzania, Uganda, Democratic Republic of Congo and Cameroon (Gillett et al., 1971). It is believed to cure coughs in East Africa (Kokwaro, 2009) and skin diseases in Central Africa (Awouafack et al., 2013a).

*Eriosema laurentii* De Wild. widely dispersed in West and Central Africa is used in this area as food and herbal medicine for many purposes such as nasopharyngeal, brain and nervous system affections, pulmonary troubles and venereal diseases (Burkill, 1985). In Cameroon, the plant is utilized for the treatment of infertility and various gynecological problems (Ateba et al., 2013).

The roots of *Eriosema chinense* Vogel (syn. *Eriosema himalaicum* H. Ohashi; *Eriosema tuberosum* (Ham.) Wang et Tang) are reported to be used as food in Northern Australia, China and North East India and against diarrhea in Meghalaya (India) (Neogi et al., 1989; Martin and Ryan, 2004; Prasad et al., 2013a). In Yunnan Province of China, the roots are used against diarrhea, orchitis, hydrophobia and as detoxifying medicine (Kunming Institute of Botany, 1979). In Thailand, the fresh roots are eaten as a tonic, and decoctions of the seeds are used for wound healing and for their diuretic properties (Thongnest et al., 2013). Such decoctions are also well-known as astringent, diuretic, tonic, against cold sweats and during delivery to promote discharge of the lochia. Decoctions additionally containing powdered pepper are given for diarrhea (Ashraf and Borthakur, 2005; Laloo and Hemalatha, 2011; Prasad et al., 2013c; Thongnest et al., 2013).

*Eriosema psoraleoides* (Lam.) G. Don. is a species widely used over Africa from Togo to Tanzania. In Tanzania, peeled or unpeeled twigs are used as chewing sticks (Khan et al., 2000), while leaves are used against health problems related to HIV/AIDS such as chronic diarrhea (Kisangau et al., 2007), impotence, erectile dysfunction, fever and malaria (Moshi et al., 2009, 2010). In several countries in Central and West Africa, leaves are applied against eye diseases (Kerharo and Bouquet, 1950; Malzy, 1954; Descoings, 1963; Wome, 1985). The roots are used in Central African Republic against ear problems (Boulesteix and Guinko, 1979), and in Democratic Republic of Congo and Burundi against sexually transmitted diseases (Staner and Boutique, 1937; Baerts and Lehmann, 1989). Eating raw roots is claimed to treat pulmonary problems in Rwanda (Durand, 1960).

Leaves of *Eriosema griseum* Baker are known as infant cures for treating parasitic diseases and associated discomforts such as stomach ache, dysentery or diarrhea in Northern parts of Ivory Coast (Koné 2005; Koné et al., 2012).

The roots of *Eriosema englerianum* Harms are traditionally used in Zimbabwe in combination with other plants such as *Vigna ungiculata* and *Terminalia sericea* to treat bilharziosis (Mmbengwa et al., 2009).

In southeastern Brazil (*Cerrado* of Minas Gerais), *Eriosema glabrum* Mart. ex Benth. is a traditional laxative, while *Eriosema benthamianum* Mart. ex Benth. and *Eriosema campestre* var. *macrophyllum* (Gear) Fortunato are reported as anti-inflammatory agents (Hirschmann and De Arias, 1990).

**FIGURE 1:**
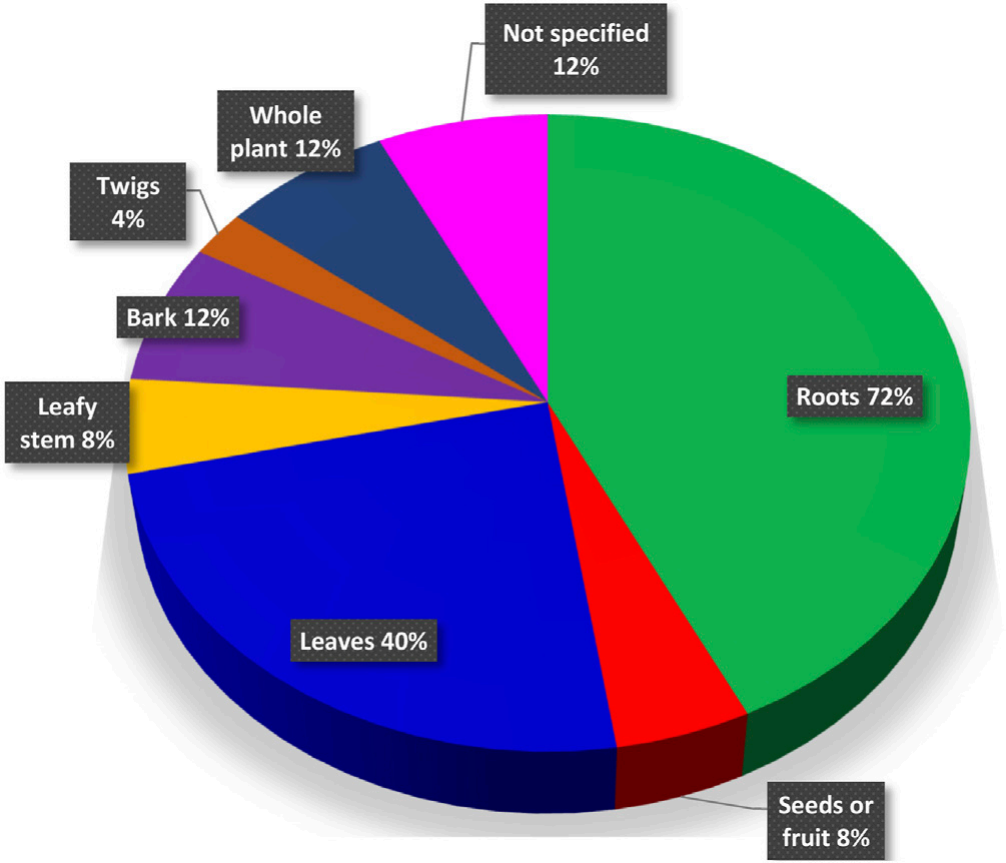
Traditionally used plant parts of *Eriosema* species.

Globally, the search of information resulted in 25 species (∼16.6% of the genus), for which written evidence of traditional uses is available. Clearly, this list cannot be exhaustive as in many traditional medicine systems a vast knowledge on medicinal plants exists as oral information only. The majority of medicinal plants used over the world are harvested from wild resources in increasing volumes. Given their integral role in basic healthcare in many developing countries, their conservation and sustainable use are a necessity. The information of the IUCN (International Union for Conservation of Nature) Red List Categories, assessing the conservation status of species, was accessed for *Eriosema* species from https://www.iucnredlist.org/fr/search?query=Eriosema&searchType=species. At the moment, only 22 *Eriosema* species (Table 2) are included in this list. Information for the vast majority of species widely used in traditional medicine such as *E. chinense*, *E. cordatum*, *E. glomeratum* and *E. psoraleoides* and others depicted in Table 1 is still missing. Only the three traditionally used species *Eriosema crinitum* (Kunth) G. Don, *Eriosema englerianum* Harms and *Eriosema montanum* Baker f. are listed, characterized by a stable population with least concern. Nevertheless, the most popular species with traditional use should be assessed to avoid a negative impact on the size of the populations. Root and whole-plant harvesting is more destructive than collecting leaves and flowers or buds (Chen et al., 2016). Among the 25 species traditionally used and reported in this review, mainly roots are utilized (18 species), followed by leaves (11 species), bark and whole plant (3 species), seeds or fruits (2 species) and twigs (1 species) (Figure 1). Rather to use roots, leaves can be an alternative to avoid the destruction and high-speed disappearing of *Eriosema* species. In case roots are unavoidable for the use, non-destructive harvest protocols such as partial-root harvest should be applied.

**TABLE 2:** The International Union for Conservation of Nature (IUCN) Red List Categories of *Eriosema* species.

Species	Scope of assessment	Population trend	Date of the last assessment	IUCN red list categories
*Eriosema adamaouense* Jacq.-Fel.	Global	Decreasing	March 25, 2011	CR
*Eriosema adami* Jacq.-Fel.	Global	Unknown	December 18, 2018	CR
*Eriosema arachnoideum* Verdc.	Global	Unknown	September 03, 2011	EN
*Eriosema arenicola* (Verdc.) Maesen & Wieringa	Global	Unknown	December 04, 2018	VU
*Eriosema benguellense* Rossberg	Global	Unknown	June 30, 2010	DD
*Eriosema betsileense* Du Puy & Labat	Global	Decreasing	September 03, 2015	EN
*Eriosema crinitum* (Kunth) G. Don	Global	Stable	August 13, 2010	LC
*Eriosema englerianum* Harms	Global	Stable	April 23, 2010	LC
*Eriosema harmsiana* Dinter	Global	Stable	April 30, 2004	LC
*Eriosema latericola* Jacq.-Fel.	Global	Unknown	December 19, 2018	EN
*Eriosema letouzeyi* Jacq.-Fel.	Global	Decreasing	March 25, 2011	VU
*Eriosema longicalyx* Grear	Global	Stable	September 16, 2010	LC
*Eriosema montanum* Baker f.	Global	Stable	September 03, 2011	LC
*Eriosema pauciflorum* Klotzsch	Global	Unknown	July 06, 2016	LC
*Eriosema procumbens* Baker	Global	Unknown	September 01, 2015	LC
*Eriosema pseudodistinctum* Verdc.	Global	Unknown	September 03, 2011	EN
*Eriosema pseudostolzii* Verdc.	Global	Unknown	September 03, 2011	VU
*Eriosema raynaliorum* Jacq.-Fel.	Global	Unknown	July 14, 2015	LC
*Eriosema spicatum* Hook.f	Global	Unknown	February 18, 2019	LC
*Eriosema triformum* Burgt	Global	Decreasing	November 11, 2016	CR
*Eriosema violaceum* (Aubl.) G. Don	Global	Stable	June 12, 2018	LC
*Eriosema chevalieri* (Harms) Hutch. & Dalziel (*syn. Rhynchosia chevalieri* Harms)	Global	Unknown	January 07, 2019	EN

CR, critically endangered; DD, data deficient; EN, endangered; LC, least concern; VU, vulnerable.

## Phytochemistry of the Genus

**FIGURE 2:**
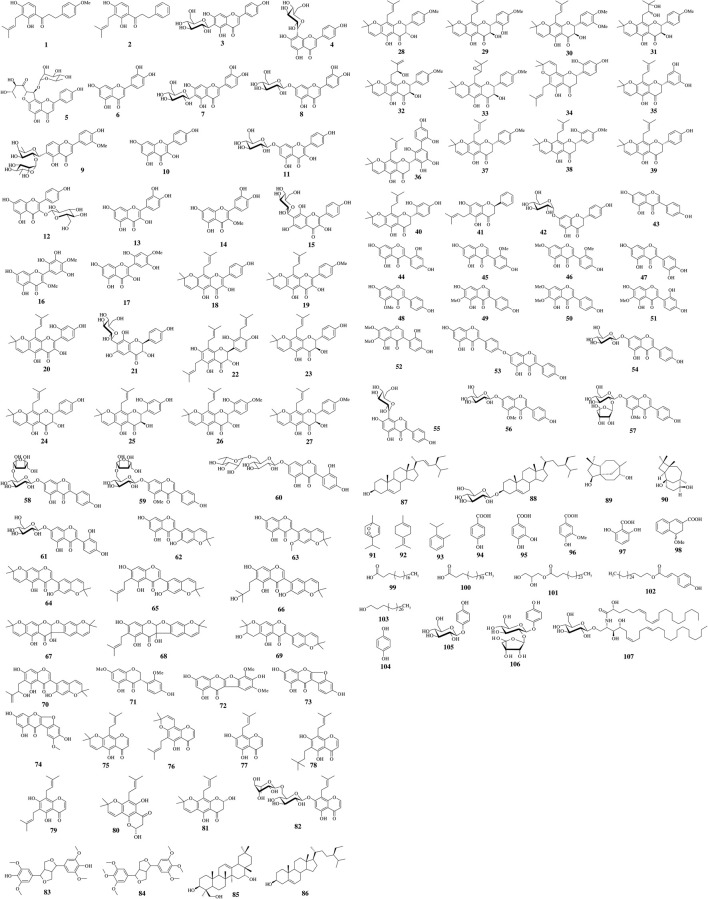
Structures of dihydrochalcones **(1**–**2)**, flavones **(3–9)**, flavonols **(10**–**20)**, dihydroflavonols **(22**–**33)**, flavanones **(34-42)**, isoflavones **(43**–**70)**, isoflavanones **(71)**, coumaronochromones **(72**–**74)**, chromones **(75–82)**, lignans **(83–84)**, terpenoids **(85–92)** and other compounds **(93–107)** from *Eriosema* spp.

Based on the ethnopharmacological applications, numerous chemical analyses of *Eriosema* species have been carried out. Compounds identified or isolated are depicted in Table 3 and Figure 2.

**TABLE 3:** Compounds isolated/identified from *Eriosema* species (the structure of compounds illustrated in Figure 2).

Chemical classes	N°	Compounds	Plant parts and species	References
**Flavonoids**				
*Dihydrochalcones*	1.	Erioschalcone a or 2ʹ,4ʹ-dihydroxy-4-methoxy-3ʹ-(*γ,γ*-dimethylallyl) dihydrochalcone (*)	Whole plant of *E. glomeratum*	Awouafack et al. (2008)
	2.	Erioschalcone B or 2ʹ,4ʹ-dihydroxy-3ʹ-(γ*,γ*-dimethylallyl)dihydrochalcone (*)	Whole plant of *E. glomeratum*	Awouafack et al. (2008)
*Flavones*	3.	Isovitexin or Apigenin-6-*C*-glucoside	Aerial parts of *E.laurentii*	Ateba et al. (2014b)
	4.	Vitexin	Twigs of *E. robustum*	Awouafack et al. (2018)
	5.	Robusflavone C	Twigs of *E. robustum*	Awouafack et al. (2018)
	6.	Luteolin	Aerial parts of *E. laurentii*	Ateba et al. (2014b)
	7.	Isoorientin or Luteolin-6-*C*-glucoside	Aerial parts of *E. laurentii*	Ateba et al. (2014b)
	8.	Luteolin-7-*O*-glucoside	Aerial parts of *E. laurentii*	Ateba et al. (2014b)
	9.	2″-*O*-α-l-Rhamnosyl-6-*C*-fucosyl-3′-*O*-methyl-luteolin	Aerial parts of *E. laurentii*	Ateba et al. (2016a)
*Flavonols*	10.	Kaempferol (*)	Roots of *E. chinense*	Thongnest et al. (2013)
			Twigs of *E. robustum*	Awouafack et al. (2018)
	11.	Kaempferol-7-*O*-*β*-d-glucopyranoside (*)	Roots of *E. chinense*	Thongnest et al. (2013)
	12.	Astragalin or Kaempferol-3-*O-β*-d-glucopyranoside (*)	Roots of *E. chinense*	Thongnest et al. (2013)
	13.	Quercetin (*)	Whole plant of *E. glomeratum*	Awouafack et al. (2008)
			Aerial parts of *E. laurentii*	Ateba et al. (2014b)
	14.	Quercetin-3-*O*-methylether	Aerial parts of *E. laurentii*	Ateba et al. (2014b)
	15.	3,4′,6,8-Tetrahydroxyflavone-7-*C*-glucoside	Aerial parts of *E. laurentii*	Ateba et al. (2016a)
	16.	Robusflavone A or 2ʹ,3ʹ,5ʹ,5,7-pentahydroxy-3,4ʹ-dimethoxyflavone (*)	Twigs of *E. robustum*	Awouafack et al. (2013a)
	17.	Robusflavone B or 2ʹ,3,5ʹ,5,7-pentahydroxy-4ʹ-methoxyflavone (*)	Twigs of *E. robustum*	Awouafack et al. (2013a)
	18.	Dehydrolupinifolinol (*)	Roots of *E.chinense*	Sutthivaiyakit et al. (2009)
	19.	Khonklonginol F or 3,5-dihydroxy-4ʹ-methoxy-6ʺ,6ʺ-dimethylpyrano (2ʺ,3ʺ:7,6)-8-(3ʹʹʹ,3ʹʹʹ-dimethylallyl)flavone (*)	Roots of *E. chinense*	Sutthivaiyakit et al. (2009)
	20.	3,5,2ʹ,4ʹ-Tetrahydroxy-6ʺ,6ʺ-dimethylpyrano (2ʺ,3ʺ:7,6)-8-(3ʹʹʹ,3ʹʹʹ-dimethylallyl)flavone (*)	Roots of *E. chinense*	Thongnest et al. (2013)
*Flavanonols or dihydroflavonols*	21.	3,4′,6,8-Tetrahydroxyflavone-7-*C*-glucoside	Aerial parts of *E. laurentii*	Ateba et al. (2016a)
	22.	(2*S*,3*S*)-6,8,3′-Triprenyl-dihydromorin or (2*S*,3*S*)-6,8,3′-triprenyl-3,5,7,2′,4′-pentahydroxyflavanone	Underground parts of *E. laurentii*	Ateba et al. (2016b)
	23.	Lupinifolinol (*)	Roots of *E. chinense*	Sutthivaiyakit et al. (2009), Thongnest et al. (2013)
	24.	3-*epi*-Lupinifolinol or (*2R,3S*)-3,5,4ʹ-trihydroxy-6ʺ,6ʺdimethylpyrano (2ʺ,3ʺ:7,6)-8-(3ʹʹʹ,3ʹʹʹ-dimethylallyl) flavanone (*)	Roots of *E. chinense*	Thongnest et al. (2013)
	25.	2ʹ-Hydroxylupinifolinol or (*2R, 3R*)-3,5,2ʹ,4ʹ-tetrahydroxy-6ʺ,6ʺ-dimethylpyrano (2ʺ,3ʺ:7,6)-8-(3ʹʹʹ,3ʹʹʹ-dimethylallyl)flavanone (*)	Roots of *E. chinense*	Thongnest et al. (2013)
	26.	3-*epi*-Khonklonginol C or (*2R,3S*)-3,5,2ʹ-trihydroxy-4ʹ-methoxy-6ʺ,6ʺ-dimethylpyrano (2ʺ,3ʺ:7,6)-8- (3ʹʹʹ,3ʹʹʹ-dimethylallyl) flavanone (*)	Roots of *E. chinense*	Thongnest et al. (2013)
	27.	Khonklonginol A or 3,5-dihydroxy-4ʹ-methoxy-6ʺ,6ʺ-dimethylpyrano (2ʺ,3ʺ:7,6)-8-(3ʹʹʹ,3ʹʺ-dimethylallyl) flavanone (*)	Roots of *E. chinense*	Thongnest et al. (2013)
				Sutthivaiyakit et al. (2009)
	28.	Khonklonginol B or 3,5-dihydroxy-4ʹ-methoxy-6ʺ,6ʺ-dimethylpyrano (2ʺ,3ʺ:7,6)-8-(3ʹʹʹ,3ʹʹʹ-dimethylallyl) flavanone (*)	Roots of *E. chinense*	Sutthivaiyakit et al. (2009)
	29.	Khonklonginol C or 3,5,2ʹ-trihydroxy-4ʹ-methoxy-6ʺ,6ʺ-dimethylpyrano (2ʺ,3ʺ:7,6)-8-(3ʹʹʹ,3ʹʹʹ-dimethylallyl) flavanone (*)	Roots of *E. chinense*	Sutthivaiyakit et al. (2009)
	30.	Khonklonginol D or 3,5-dihydroxy-3ʹ,4ʹ-dimethoxy-6ʺ,6ʺ-dimethylpyrano (2ʺ, 3ʺ: 7,6)-8-(3ʹʹʹ,3ʹʹʹ-dimethylallyl)flavanone (*)	Roots of *E. chinense*	Sutthivaiyakit et al. (2009)
	31.	Khonklonginol E or 3,5-dihydroxy-4′-methoxy-6ʺ,6ʺ-dimethylpyrano (2ʺ,3ʺ:7,6)-8-(3ʹʹʹ,3ʹʹʹ-dimethyl-2ʹʹʹ,3ʹʹʹ-dihydroxypropyl) flavanone (*)	Roots of *E. chinense*	Sutthivaiyakit et al. (2009)
	32.	(*2R*,*3R*,*2‴R*)-3,5,2‴-Trihydroxy-4′-methoxy-6″,6″-dimethylpyrano (2″,3″:7,6)-8-(3‴-methylbut-3‴-enyl) flavanone (*)	Roots of *E. chinense*	Thongnest et al. (2013)
	33.	2ʹʹʹ,3ʹʹʹ-Epoxykhonklonginol A or (*2R,3R*)-3,5-dihydroxy-4ʹ-methoxy-6ʺ,6ʺ-dimethylpyrano (2ʺ,3ʺ:7,6)-8-(2ʹʹʹ,3ʹʹʹ-epoxy-3ʹʹʹ-methylbutyl)flavanone (*)	Roots of *E. chinense*	Thongnest et al. (2013)
*Flavanones*	34.	Eriosemaone A (*)	Roots of *E. tuberosum*	Ma et al. (1995)
			Roots of *E. chinense*	Sutthivaiyakit et al. (2009)
	35.	Eriosemaone B (*)	Roots of *E. tuberosum*	Ma et al. (1995)
	36.	Eriosemaone C (*)	Roots of *E. tuberosum*	Ma et al. (1995)
	37.	Khonklonginol G or 5-hydroxy-4ʹ-methoxy-6ʺ,6ʺ-dimethylpyrano (2ʺ,3ʺ:7,6)-8-(3ʹʹʹ,3ʹʹʹ-dimethylallyl) flavanone (*)	Roots of *E. chinense*	Sutthivaiyakit et al. (2009)
	38.	Khonklonginol H or 5,2ʹ-dihydroxy-4ʹ-methoxy-6ʺ,6ʺ-dimethylpyrano (2ʺ,3ʺ:7,6)-8-(3ʹʹʹ,3ʹʹʹ-dimethylallyl) flavanone (*)	Roots of *E. chinense*	Sutthivaiyakit et al. (2009)
	39.	Lupinifolin (*)	Roots of *E. tuberosum*	Ma et al. (1996b)
			Roots of *E. chinense*	Prasad et al. (2013a)
				Sutthivaiyakit et al. (2009)
	40.	Flemichin D (*)	Roots of *E. tuberosum*	Ma et al. (2015)
			Roots of *E. chinense*	Sutthivaiyakit et al. (2009), Thongnest et al. (2013)
	41.	6-Prenyl pinocembrin (*)	Twigs of *E. robustum*	Awouafack et al. (2013a)
	42.	Prunin	Twigs of *E. robustum*	Awouafack et al. (2018)
*Isoflavones*	43.	Genistein (*)	Roots and twigs of *E. tuberosum*	Ma et al. (1998), Awouafack et al. (2018)
			Roots of *E. chinense*	Thongnest et al. (2013)
			Aerial and underground parts of *E. laurentii*	Ateba et al. (2013), Ateba et al. (2016b)
	44.	2′-Hydroxygenistein	Aerial and underground parts of *E. laurentii*	Ateba et al. (2014b), Ateba et al. (2016b)
	45.	5,7,4′-Trihdroxy-2′-methoxyisoflavone	Roots of *E. psoraleoides*	Wanyama (2010)
	46.	4′,5-Dihydroxy-2′,7-dimethoxyisoflavone	Roots of *E. psoraleoides*	Wanyama (2010)
	47.	Isoluteolin (*)	Whole plant of *E. glomeratum*	Awouafack et al. (2008)
	48.	5-*O*-Methylgenistein (*)	Roots of *E. tuberosum*	Ma et al. (1998)
	49.	Tectorigenin (*)	Roots of *E. chinense*	Thongnest et al. (2013)
	50.	7-*O*-Methyltectorigenin (*)	Roots of *E. chinense*	Thongnest et al. (2013)
	51.	5,7,2′,4ʹ-Tetrahydroxy-6-methoxyisoflavone (*)	Roots of *E. chinense*	Thongnest et al. (2013)
	52.	6,7-Dimethoxy-5,2ʹ,4ʹ-trihydroxyisoflavone (*)	Roots of *E. chinense*	Thongnest et al. (2013)
	53.	4′,7″-Bisgenistein	Roots of *E. psoraleoides*	Wanyama (2010)
	54.	Genistin or genistein-7-*O-β*-*d*-glucopyranoside (*)	Roots of *E. tuberosum*	Ma et al. (1998)
			Aerial and underground parts of *E. laurentii*	Ateba et al. (2013), Ateba et al. (2016b)
			Roots of *E. chinense*	Thongnest et al. (2013)
	55.	Genistein-8-*C*-glucopyranoside	Aerial parts of *E. laurentii*	Ateba et al. (2016a)
	56.	5-*O*-Methylgenistein-7-*O*-*β*-d-glucopyranoside (*)	Roots of *E. tuberosum*	Ma et al. (1998)
	57.	Eriosemaside C or 5-*O*-Methylgenistein-7-*O*-*β*-d-apiofuranosyl-(1→2)-*O*-*β*-d- glucopyranoside (*)	Roots of *E. tuberosum*	Ma et al. (1999)
	58.	Genistein-7-*O*-*β*-D-apiofuranosyl-(1→6)-*O*-*β*-d-glucopyranoside (*)	Roots of *E. tuberosum*	Ma et al. (1998)
	59.	5-*O*-Methylgenistein-7-*O*-*β*-d-apiofuranosyl-(1→6)-*O*-*β*-d-glucopyranoside (*)	Roots of *E. tuberosum*	Ma et al. (1998)
	60.	Sphaerobioside (*)	Roots of *E. tuberosum*	Ma et al. (1998)
	61.	2′-Hydroxygenistein-7-*O*-glucopyranoside	Aerial parts of *E. laurentii*	Ateba et al. (2016a)
	62.	Eriosemaone D or 5,7,2′-trihydroxy-6″,6″-dimethylpyrano (2″,3″:4′,5′) isoflavone (*)	Roots of *E. tuberosum*	Ma et al. (1995)
			Underground parts of *E. laurentii*	Ateba et al. (2016b)
	63.	2′-*O*-Methyl-eriosemaone D or 5,7-dihydroxy-2′-methoxy-6″,6″-dimethyl-pyrano (2″,3″:4′,5′) isoflavone	Underground parts of *E. laurentii*	Ateba et al. (2016b)
	64.	Kraussianone 1 or 5,2ʹ-dihydroxy-[(6ʺ,6ʺ-dimethylpyrano (2ʺ,3ʺ:4ʹ,5ʹ)] [(6ʹʹʹ,6ʹʹʹ-dimethylpyrano (2ʹʹʹ,3ʹʹʹ:7,6)] isoflavone (*)	Rootstock of *E. kraussianum*	Drewes et al. (2002)
				Ojewole et al. (2006)
	65	Kraussianone 2 (*)	Rootstock of *E. kraussianum*	Drewes et al. (2002)
				Ojewole et al. (2006)
				Ramesar et al. (2012)
	66.	Kraussianone 3 (*)	Rootstock of *E. kraussianum*	Drewes et al. (2002)
	67.	Kraussianone 4 (*)	Rootstock of *E. kraussianum*	Drewes et al. (2002)
	68.	Kraussianone 5 (*)	Rootstock of *E. kraussianum*	Drewes et al. (2002)
	69.	Kraussianone 6 (*)	Rootstock of *E. kraussianum*	Drewes et al. (2004)
	70.	Kraussianone 7 (*)	Rootstock of *E. kraussianum*	Drewes et al. (2004)
*Isoflavanones*	71.	Cajanol (*)	Roots of *E. chinense*	Thongnest et al. (2013)
*Coumaronochromones*	72.	Eriocoumaronochromone	Twigs of *E. robustum*	Awouafack et al. (2018)
	73.	Lupinalbin A or 5.7,4ʹ-trihydroxy-coumaronochromone	Aerial and underground parts of *E. laurentii*	Ateba et al. (2014b), Ateba et al. (2016b)
			Roots of *E. psoraleoides*	Wanyama (2010)
	74.	Desmoxyphyllin A or 5,7,4′-trihydroxy-5′-methoxycoumaronochromone	Twigs of *E. robustum*	Awouafack et al. (2018)
**Chromones**				
	75.	Eriosematin or 5-hydroxy-8-*γ,γ*-dimethylallyl-6ʺ,6ʺ-dimethylpyrano (3ʺ,2ʺ:6,7) chromone	Roots of *E. tuberosum*	Ma et al. (1995)
	76.	Iso-eriosematin or 5-hydroxy-6-*γ,γ*-dimethylallyl-6ʹ,6ʹ-dimethyl-pyrano (2ʹ, 3ʹ: 7,8) chromone	Roots of *E. tuberosum*	Ma et al. (1996a)
	77.	Eriosematin A or 5,7-dihydroxy-8-*γ,γ*-dimethylallyl chromone	Roots of *E. tuberosum*	Ma et al. (1996a)
	78.	Eriosematin B or 5,7-dihydroxy-6-(3-hydroxy-3,3-dimethylbutyl)-8-*γ,γ*-dimethylallyl chromone	Roots of *E. tuberosum*	Ma et al. (1996a)
	79.	Eriosematin C or 5,7-dihydroxy-6-*γ,γ*-dimethylallyl-8-*γ,γ*-dimethylallyl chromone	Roots of *E. tuberosum*	Ma et al. (1996a)
	80.	Eriosematin D or 2,5-dihydroxy-6-*γ,γ*-dimethylallyl-6ʺ,6ʺ-dimethylpyrano (2ʺ,3ʺ:7,8) chromone	Roots of *E. tuberosum*	Ma et al. (1996b)
	81.	Eriosematin E or 2,5-dihydroxy-8-*γ,γ*-dimethylallyl-6ʺ,6ʺ-dimethylpyrano (2ʺ,3ʺ:7,6) chromone	Roots of *E. tuberosum*	Ma et al. (1996b)
			Roots of *E. chinense*	Prasad et al. (2017)
	82.	Eriosemaside A or 5,7-dihydroxy-8-*γ,γ*-dimethylallylchromone-7-*O*-rutinoside	Roots of *E. tuberosum*	Ma et al. (1999)
**Lignans**				
	83.	Syringaresinol	Aerial parts of *E. laurentii*	Ateba et al. (2016a)
	84.	Yangambin	Roots of *E. chinense*	Sutthivaiyakit et al. (2009)
**Terpenoids**				
*Triterpenoid*	85.	Olean-12- ene-3,16,23-triol	Whole plant of *E. glomeratum*	Awouafack et al. (2008)
*Sterols*	86.	*β*-Sitosterol	Twigs of *E. robustum*	Awouafack et al. (2013a)
			Roots of *E. chinense*	Thongnest et al. (2013)
	87.	Stigmasterol	Twigs of *E. robustum*	Awouafack et al. (2013a), Thongnest et al. (201a)
			Roots of *E. chinense*	
	88.	β-Sitosterol-3-*O-β*-d-glucopyranoside	Whole plant of *E. glomeratum*	Awouafack et al. (2008)
			Twigs of *E. robustum*	Awouafack et al. (2013a)
*Sesquiterpenes*	89.	Clovanediol	Roots of *E. chinense*	Thongnest et al. (2013)
	90.	Caryolane-1,9-diol	Roots of *E. chinense*	Thongnest et al. (2013)
*Monoterpenes*	91.	Ascaridole	Leaves of *E. englerianum*	Mmbengwa et al. (2009)
	92.	Terpinolene	Leaves of *E. englerianum*	Mmbengwa et al. (2009)
**Other compounds**				
*Toluene derivatives*	93.	*O*-Cymene	Leaves of *E. englerianum*	Mmbengwa et al. (2009)
*Benzoic acid Derivatives*	94.	4-Hydroxybenzoic acid or *p*-Hydroxybenzoic acid	Roots of *E. tuberosum*	Ma et al. (1999)
			Twigs of *E. robustum*	Awouafack et al. (2018)
			Aerial parts of *E. laurentii*	Ateba et al. (2016a)
	95.	3,4-Dihydroxybenzoic acid	Aerial parts of *E. laurentii*	Ateba et al. (2016a)
	96.	Vanillic acid	Roots of *E. tuberosum*	Ma et al. (1999)
	97.	2,6-Dihydroxybenzoic acid	Aerial parts of *E. laurentii*	Ateba et al. (2016a)
	98.	Eriosematin F or 3,4 dihydro-4-methoxy-naphthalene-2-carboxylic acid	Roots of *E. tuberosum*	Ma et al. (1999)
*Fatty acids*	99.	Eicosanoic acid or arachidic acid	Twigs of *E. robustum*	Awouafack et al. (2013a)
	100.	Tetratriacontanoic acid	Whole plant of *E. glomeratum*	Awouafack et al. (2008)
*Glycerol derivative*	101.	1-*O*-Heptatriacontanoyl glycerol	Twigs of *E. robustum*	Awouafack et al. (2013a)
*Coumarate*	102.	Octaeicosanyl-*trans*-*p*-coumarate	Roots of *E. chinense*	Thongnest et al. (2013)
*Fatty alcohol*	103.	Triacontanol	Whole plant of *E. glomeratum*	Awouafack et al. (2008)
*Phenols*	104.	Hydroquinone	Roots of *E. tuberosum*	Ma et al. (1999)
	105.	Arbutin	Roots of *E. tuberosum*	Ma et al. (1999)
	106.	Eriosemaside B or 4-hydroxyphenyl *β*-d-apiofuranosyl-(1→2)-*O*-*β*-d-glucopyranoside	Roots of *E. tuberosum*	Ma et al. (1999)
*Cerebroside*	107.	Orostachyscerebroside A	Twigs of *E. robustum*	Awouafack et al. (2013a)

* Flavonoids compiled by Awouafack et al. (2015).

### Flavonoids

In their review, Awouafack et al. (2015) compiled a total of 52 natural flavonoids (indicated by (*) in Table 3) from five *Eriosema* species (*E. chinense*, *E. tuberosum*, *E. kraussianum*, *E. glomeratum* and *E. robustum*), including dihydrochalcones (**1–2**), flavonols (**10–13**, **16–20**), flavanonols or dihydroflavonols (**23–33**), flavanones (**34–41**), isoflavones (**43**, **47–52**, **54**, **56–60**, **62**, **64** **–70**) and one isoflavanone (**71**). Flavonoids/isoflavonoids not recorded in that review and/or from different *Eriosema* species are mentioned below.

In a dereplication approach, the flavones isovitexin (**3**), luteolin (**6**), isoorientin (**7**) and luteolin-7-*O*-glucoside (**8**) were unambiguously identified in a methanol extract of the aerial parts of *E. laurentii* (Ateba et al., 2014b) and 2″-*O*-α-l-rhamnosyl-6-*C*-fucosyl-3′-*O*-methyl-luteolin (**9**) was isolated from the same extract (Ateba et al., 2016a). Vitexin (**4**) and robusflavone C (**5**) were extracted from an ethanol fraction of twigs of *E. robustum* (Awouafack et al., 2018).

The flavonol kaempferol (**10**) was isolated from roots of *E. chinense* (Thongnest et al., 2013) and twigs of *E. robustum* (Awouafack et al., 2018).

Quercetin (**13**) and quercetin-3-*O*-methylether (**14**) were identified in a methanol extract of the aerial parts of *E. laurentii* (Ateba et al., 2014b), while 3,4′,6,8-tetrahydroxyflavone-7-*C*-glucoside (**15**) from that extract was identified for the first time in the genus (Ateba et al., 2016a).

The flavanonols 3,4′,6,8-tetrahydroxyflavanone-7-*C*-glucoside (**21**) and (2*S*,3*S*)-6,8,3′-triprenyl-dihydromorin (**22**) from the methanol extracts of the aerial (Ateba et al., 2016a) and underground (Ateba et al., 2016b) parts of *E. laurentii,* respectively, were also identified for the first time in the genus. Among flavanones, prunin (**42**) was isolated from the twigs of *E. robustum* (Awouafack et al., 2018).

The isoflavones, genistein (**43**), 2ʹ-hydroxygenistein (**44**), genistin (**54**), genistein-8-*C*-glucoside (**55**), 2ʹ-hydroxygenistein-7-*O*-glucoside (**61**), eriosemaone D (**62**) and the new compound 2ʹ-*O*-methyl-eriosemaone D (**63**) were identified and/or isolated from *E. laurentii* (Ateba et al., 2013; 2014b; 2016a; 2016b). Compound **43** was also obtained from the EtOAc-soluble portion of an EtOH extract of the twigs of *E. robustum* (Awouafack et al., 2018). 5,7,4′-trihdroxy-2′-methoxyisoflavone (**45**), 4′,5-dihydroxy-2′,7-dimethoxyisoflavone (**46**) and 4′,7″-bisgenistein (**53**) were obtained from a dichloromethane/methanol (1:1 *v/v*) root extract of *E. psoraleoides* (Wanyama, 2010).

The coumaronochromone named eriocoumaronochromone (**72**) was isolated for the first time from an EtOH extract of the twigs of *E. robustum*, along with desmoxyphyllin A (**74**) (Awouafack et al., 2018). Lupinalbin A (**73**) was proven in methanol extracts of the aerial and underground parts of *E. laurentii* (Ateba et al., 2014b; 2016b) and in a dichloromethane/methanol (1:1 *v/v*) root extract of *E. psoraleoides* (Wanyama, 2010).

### Chromones

All chromones known in the genus including eriosematin (**75**), iso-eriosematin (**76**), eriosematin A-E (**77–81**) and eriosemaside A (**82**) were obtained from the roots of *E. tuberosum* (Ma et al., 1995; 1996a; 1996b, 1999). Compound **81** was also identified in an ethanol root extract of *E. chinense* (Prasad et al., 2017).

### Lignans

Syringaresinol **(83**) was isolated from the aerial parts of *E. laurentii* (Ateba et al., 2016a), while yangambin **(84**) was found in the roots of *E. chinense* (Sutthivaiyakit et al., 2009).

### Terpenoids

The investigation of a dichloromethane/methanol (1:1, v/v) extract of the whole plant of *E. glomeratum* gave the triterpenoid olean-12-ene-3,16,23-triol **(85**) and the sterol 3-*O-β-* *d*-sitosterol glucopyranoside **(88**) (Awouafack et al., 2008). The sterols β-sitosterol **(86**) and stigmasterol **(87**) were reported from an ethanol extract of the twigs of *E. robustum* (Awouafack et al., 2013a) and roots of *E. chinense* (Thongnest et al., 2013). The sesquiterpenes clovanediol **(89**) and caryolane-1,9-diol (**90**) were obtained from roots of *E. chinense* (Thongnest et al., 2013), while the monoterpenes ascaridole **(91**) and terpinolene **(92**) have been isolated from the essential oil of leaves of *E. englerianum* (Mmbengwa et al., 2009).

### Other Compounds

*O*-cymene **(93**) was obtained as a major phytoconstituent of the essential oil from fresh leaves of *E. englerianum*. 4-Hydroxybenzoic acid **(94**) was isolated from roots of *E. tuberosum* (Ma et al., 1999) and twigs of *E. robustum* (Awouafack et al., 2018) and from the aerial parts of *E. laurentii* along with 3,4-dihydroxybenzoic acid **(95**) and 2,6-dihydroxybenzoic acid **(97**) (Ateba et al., 2016a). Vanillic acid **(96**) and eriosematin F **(98**) were found in roots of *E. tuberosum* (Ma et al., 1999). Eicosanoic acid **(99**) was isolated from twigs of *E. robustum* (Awouafack et al., 2013a) and tetratriacontanoic acid (**100**) from a whole plant extract of *E. glomeratum* (Awouafack et al., 2008). The glycerol derivative 1-*O*-heptatriacontanoyl glycerol (**101**) was isolated from twigs of *E. robustum* (Awouafack et al., 2013a), while octaeicosanyl-trans-*p*-coumarate (**102**) was obtained from the roots of *E. chinense* (Thongnest et al., 2013). The fatty alcohol triacontanol (**103**) was isolated from *E. glomeratum*. Three phenols identified as hydroquinone (**104**), arbutin (**105**) and eriosemaside B (**106**) were isolated from the roots of *E. tuberosum* (Ma et al., 1999). Twigs of *E. robustum* contained orostachyscerebroside A (**107**) (Awouafack et al., 2013a).

**FIGURE 3:**
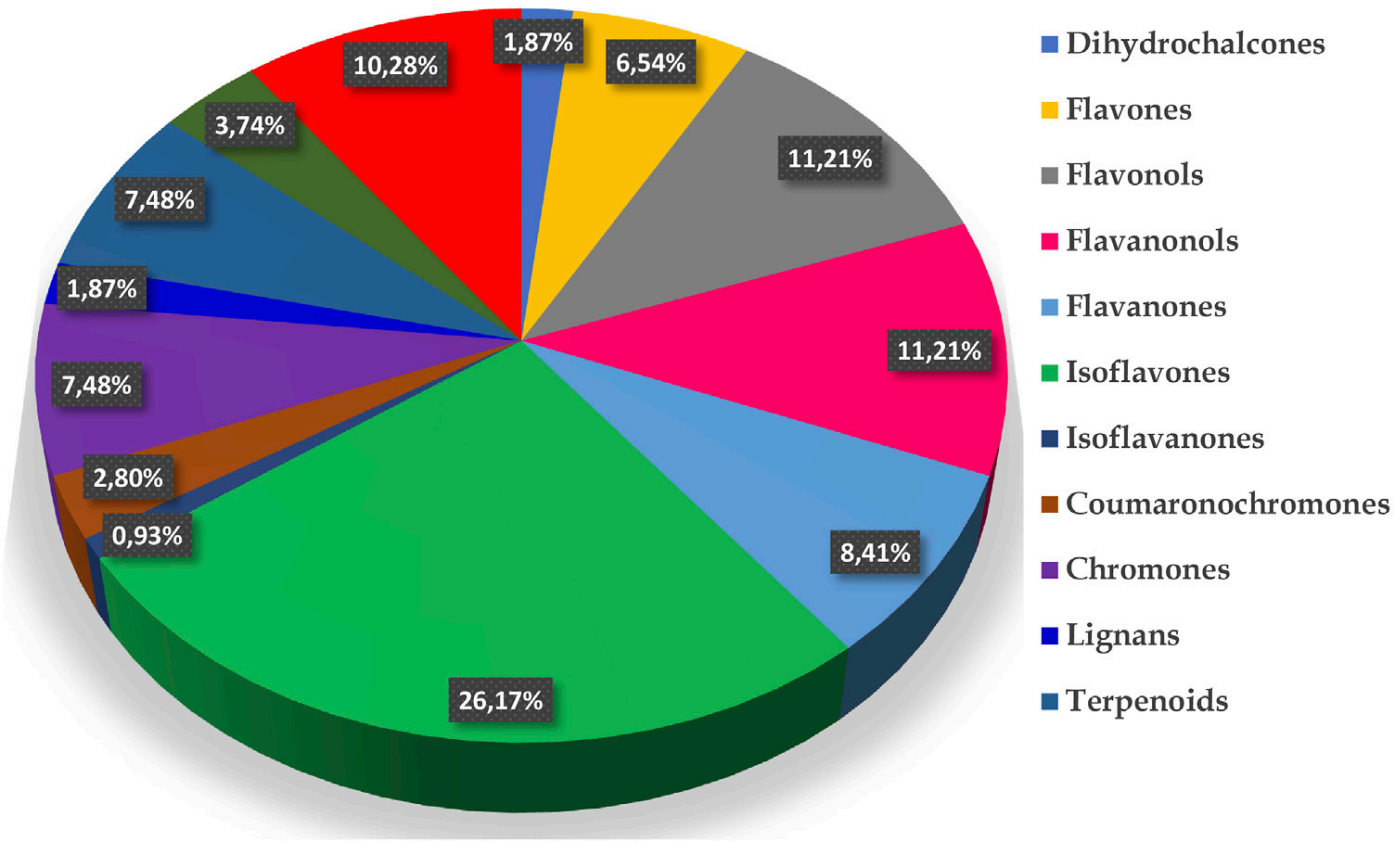
Chemical diversity of *Eriosema* genus.

Globally the phytochemical investigation of the *Eriosema* genus led to the isolation and/or identification of 107 compounds from only eight species (5.3% of the genus) out of *ca.* 150 (*E. chinense*, *E. englerianum*, *E. glomeratum*, *E. kraussianum*, *E. laurentii*, *E. robustum*, *E. tuberosum* and *E. psoraleoides*). Isolated and/or identified constituents are mostly isoflavones (26.2%), flavonols (11.2%), flavanonols (11.2%), flavanones (8.4%) and chromones (7.5%) (Figure 3). Awouafack et al. (2015) documented flavonoids from 5 *Eriosema* species and concluded that the genus represents a rich source of flavonoids, mostly isoflavones which could be considered as markers for the genus and have chemotaxonomic significance. However, given the low number of species investigated until now, the deduction of a chemotaxonomic significance of some of the components seems premature.

## Pharmacology

Based on their ethnopharmacological use, *Eriosema* species aroused the interest of the scientific community. In line with this, many studies have been carried out aiming at evaluating or confirming the therapeutic potential of species from this genus. As depicted in Table 4, extracts and constituents from *Eriosema* species are shown to be responsible for a wide range of pharmacological activities such as aphrodisiac, anti-diarrheal, anti-oxidant, anti-microbial, anti-diabetic/hypoglycemic and estrogenic properties.

**TABLE 4:** Pharmacological properties of *Eriosema* spp.

Pharmacological activities	*Eriosema* species	Parts/extracts	Model	Dosage/concentration and route of administration in the animal studies	Results	References
Anticancer	*E. chinense*	Roots/hexane extract	*In vitro*: MTT assay Small-cell lung (NCI-H187), oral epidermal carcinoma (KB) human cancer cells		Moderate activity (IC_50_:12.0 μg/ml) against small-cell lung and oral epidermal carcinoma (IC_50_: 9.9 μg/ml) human cells	Sutthivaiyakit et al. (2009)
Anti-oxidant	*E.englerianum*	Leaves/essential oil	*In vitro*: β-carotene bleaching assay		Color retention zone of 14.4 mm	Mmbengwa et al. (2009)
	*E. chinense*	Roots/EtOH extract	*In vitro*: PFRAP assay		Reducing power of 0.263 μg/ml	Prasad et al. (2013b)
			*In vitro*: DPPH, H_2_O_2_ and nitric oxide radical scavenging assays		Scavenging activities of differing degree with IC_50_ values of 146, 221, 233 and 170 μg/ml, respectively	
	*E. robustum*	Twigs/EtOH extract	DPPH assay		Scavenging activity with IC_50_ ≥ 1.13 mg/ml	Awouafack et al. (2013b)
Estrogenic	*E. laurentii*	Aerial parts/MeOH extract	*In vitro*: ERα yeast two-hybrid assay	1–83 μg/ml	Concentration-dependent β-galactosidase activity	Ateba et al. (2013)
			*In vivo*: Ovariectomized Wistar rats *In vivo*: Ovariectomized Wistar rats	50, 100 and 200 mg/kg daily for 3 days and 9 weeks p.o	Vaginal stratification and cornification, no effect on uterus	Ateba et al. (2013)
		Underground parts/MeOH extract		50, 100 and 200 mg/kg daily for 3 days p.o	No effect on uterus and vagina	Ateba (2014c)
Aryl hydrocarbon agonistic	*E. laurentii*	Aerial parts/MeOH extract	*In vitro*: AhR yeast β-galactosidase assay	1–83 μg/ml	Concentration-dependent AhR β-galactosidase activity	Ateba et al. (2013)
		Underground parts/MeOH extract		1–125 μg/ml		Ateba et al. (2016b)
Anti-osteoporosis	*E. laurentii*	Aerial parts/MeOH extract	*In vivo*: Ovariectomized Wistar rats	50, 100 and 200 mg/kg daily for 9 weeks p.o	↑ dry femur weight, ↑ serum and femur calcium levels ↓ serum inorganic phosphorus levels, ↑ femur inorganic phosphorus levels	Ateba et al. (2013)
Hypoglycemic and anti-diabetic	*E. kraussianum*	Rootstock/hydro-alcoholic extract	*In vivo*: normal Wistar and streptozotocin-induced diabetic Wistar rats	40, 80, 160 and 320 mg/kg p.o. (single dose)	↓ Fasting blood glucose levels in dose-dependent manner from 2 to 8 h after treatment with peak effects 4 h after	Ojewole et al. (2007)
	*E. psoraleoides*	Leaves/H_2_O and EtOH extracts	*In vivo*: alloxan-induced diabetic Wistar rats	200 and 400 mg/kg daily for 7 days p.o	↓ Fasting blood glucose levels from day 6	Nduka et al. (2018), Nduka et al. (2019)
		Leaves/*n*-hexane extract, *n*-hexane-DCM and DCM fractions	*In vivo*: alloxan-induced diabetic Wistar rats	200 mg/kg daily for 7 days p.o	↓ Fasting blood glucose levels from day 2–7	Elechi et al. (2019)
Cardiovascular protection	*E. laurentii*	Aerial parts/MeOH extract	*In vivo*: postmenopause-like model (ovariectomized Wistar rats)	50, 100 and 200 mg/kg daily for 9 weeks p.o	↓Total cholesterol (TC) and LDL levels ↑ HDL ↓TC/HDL and LDL/HDL ratios, atherogenic plasma index	Ateba et al. (2013)
Acetylcholinesterase inhibition	*E. cordatum*	Roots/H_2_O extract	*In vitro*: Acetylcholinesterase inhibition		Low activity (IC_50_ of 757 μg/ml)	Ndhlala et al. (2011)
Anti-microbial	*Eriosema campestre* var. *campestre*	Leaves/MeOH extract	*In vitro* *Trichophyton rubrum* *Trichophyton mentagrophytes* *Microsporum gypseum* *Epidermophyton floccosum* *Candida albicans* ATCC 90028 *Candida krusei* ATCC 6258 *Candida glabrata* HCCGL01 *Candida tropicalis* ATCC 750 *Candida parapsilosis* ATCC22019	500 μg/ml	Fungistatic toward *T. rubrum*, *T. mentagrophytes* and *E. floccosum*. Inactive against *M. gypseum* and all tested *Candida* strains	de Morais et al. (2017)
	*Eriosema campestre* var. *macrophyllum*	Leaf/MeOH extract	*In vitro* *Trichophyton rubrum* *Trichophyton mentagrophytes* *Microsporum gypseum* *Epidermophyton floccosum* *Candida albicans* ATCC 90028 *Candida krusei* ATCC 6258 *Candida glabrata* HCCGL01 *Candida tropicalis* ATCC 750 *Candida parapsilosis* ATCC22019	500 μg/ml	Fungistatic toward *T. mentagrophytes* and *E. floccosum*. Inactive against *T. rubrum*, *M. gypseum* and all tested *Candida* strains	de Morais et al. (2017)
	*E. cordatum*	Roots/petroleum ether, DCM, EtOH and H_2_O extracts	*In vitro* *Staphylococcus aureus* *Bacillus subtilis* *Escherichia coli* *Klebsiella pneumonia*		MIC of 390 μg/ml with ethanolic extract MIC of 195 μg/ml against *S. aureus* and *B. subtilis*, and of 390 μg/ml against *E. coli* and *K. pneumoniae* with dichloromethane extract Petroleum ether and H_2_O extracts not active	Ndhlala et al. (2011)
	*E. chinense*	Roots/EtOH extract	*In vitro* *Escherichia coli* ATCC25922, *Shigella flexneri* ATCC12022 *Pseudomonas aeruginosa* ATCC27893 *Staphylococcus aureus* ATCC25323 *Salmonella typhi* *Shigella dysenteriae* *Proteus vulgaris* *Klebsiella pneumoniae* *Shigella boydii* *Bacillus cereus* *Enterococcus faecalis*		MICs >195 μg/ml	Prasad et al. (2013)
	*E. englerianum*	Leaves/essential oil	*In vitro* *Staphylococcus aureus* *Escherichia coli* *Pseudomonas aeruginosa * *Bacillus subtilis* *Klebsiella pneumoniae* *Proteus vulgaris* *Clostridium sporogenes* *Acinetobacter calcoaceticus* *Candida albicans* *Aspergillus niger* *Aspergillus flavus*	1, 2, 5 and 10 μL/ml	IZ ≥ 7 mm at 5 μL/ml (*A. calcoaceticus*) and 10 μL/ml (*C. sporogenes* and *A. calcoaceticus*) ↓ growth of *C. albicans* (24.2–42.6%), *Aspergillus niger* (34.7–49.7%) and *Aspergillus flavus* (27.5–39.3%)	Mmbengwa et al. (2009)
	*Eriosema glabrum*	Leaves/MeOH extract	*In vitro* *Trichophyton rubrum* *Trichophyton mentagrophytes* *Microsporum gypseum* *Epidermophyton floccosum* *Candida albicans* ATCC 90028 *Candida krusei* ATCC 6258 *Candida glabrata* HCCGL01 *Candida tropicalis* ATCC 750 *Candida parapsilosis* ATCC22019	500 μg/ml	Fungicidal toward *T. mentagrophytes* and *E. floccosum*. Inactive against *T. rubrum*, *M. gypseum* and all tested *Candida* strains	de Morais et al. (2017)
	*E. glomeratum*	Stem and leaves/MeOH-DCM extract	*In vitro* *Staphylococcus aureus* ATCC25923 *Klebsiella pneumonia* ATCC13883 *Morexella catarrhalis* ATCC 14468 *Mycobacterium smegmatis* ATCC23246 *Mycobacterium aurum* NCTC10437		Stem extract: MICs of 500, 1000, 1000, 65, and 50 μg/ml, respectively Leaf extract: MICs of 1,000, 500, 1,000,130 and 1,000 μg/ml, respectively	Fomogne-Fodjo et al. (2014)
	*Eriosema heterophyllum*	Leaves/MeOH extract	*In vitro* *Trichophyton rubrum* *Trichophyton mentagrophytes* *Microsporum gypseum* *Epidermophyton floccosum* *Candida albicans* ATCC 90028 *Candida krusei* ATCC 6258 *Candida glabrata* HCCGL01 *Candida tropicalis* ATCC 750 *Candida parapsilosis* ATCC22019	500 μg/ml	Fungistatic toward *T. rubrum*, *T. mentagrophytes* and *M. gypseum*. Fungicidal against *E. floccosum*. Inactive against, and all tested *Candida* strains	de Morais et al. (2017)
	*Eriosema longifolium*	Leaves/MeOH extract	*In vitro* *Trichophyton rubrum* *Trichophyton mentagrophytes* *Microsporum gypseum* *Epidermophyton floccosum* *Candida albicans* ATCC 90028 *Candida krusei* ATCC 6258 *Candida glabrata* HCCGL01 *Candida tropicalis* ATCC 750 *Candida parapsilosis* ATCC22019	500 μg/ml	Inactive	de Morais et al. (2017)
	*E. montanum*	Leaves/polar fraction of an EtOH extract	*In vitro* *Bacillus cereus* ATCC 14579, *Enterobacter cloacae* ATCC 13047 *Escherichia coli* ATCC 8739 *Klebsiella pneumoniae* ATCC 13883 *Mycobacterium fortuitum* ATCC 6841 *Proteus vulgaris* ATCC 13315 *Pseudomonas aeruginosa* ATCC 15442 *Salmonella typhimurium* ATCC 13311 *Staphylococcus aureus* ATCC 6538 *Streptococcus pyogenes* ATCC 12344 *Candida albicans* ATCC 10231 *Trichophyton rubrum* RV 58125, *Epidermophyton floccosum* RV 71625 *Microsporum canis* RV 66973		MIC > 750 μg/ml	Cos et al. (2002b)
	*E. psoraleoides*	Bark and wood of twigs/MeOH extract	*In vitro* *Streptococcus mutans* HG982 *Actinomyces viscosus* HG485 *Candida albicans* HG392		Inactive (MIC ≥1.25 mg/ml)	Khan et al. (2000)
		Stem bark/MeOH extract	*In vitro* *Candida albicans*		Inactive	Runyoro et al. (2006)
		Leaves/MeOH extract and its n-hexane and ethyl acetate fractions	*In vitro* *Escherichia coli* ATCC35219, *Staphylococcus aureus* ATCC25923 Clinical isolates of *Staphylococcus aureus* *Proteus vulgaris* *Klebsiella erogenes* *Pseudomonas aeruginosa* *Escherichia coli*		Inactive (MIC >1.26 mg/ml, except n-hexane fraction (MIC of 150 μg/ml) toward *S. aureus* ATCC	Elechi and Igboh (2017)
	*E. robustum*	Twigs/EtOH extract	*In vitro* *Staphylococcus aureus* ATCC29213 *Enterococcus faecalis* ATCC29212 *Pseudomonas aeruginosa* ATCC27853 *Escherichia coli* ATCC25922 *Candida albicans* ATCC10231 *Candida albicans* *Cryptococcus neoformans* *Aspergillus fumigatus*		MIC of 80 μg/ml toward *S. aureus* *E. faecalis* and *E. coli* MIC of 310 μg/ml toward *P. aeruginosa* MIC of 160 μg/ml toward *A. fumigatus* MIC of 630 μg/ml against *C. neoformans* MIC of 1250 μg/ml) toward *C. albicans* and *C. albicans* ATCC	Awouafack et al. (2013a)
	*Eriosema tacuaremboense*	Leaves/MeOH extract	*In vitro* *Trichophyton rubrum* *Trichophyton mentagrophytes* *Microsporum gypseum* *Epidermophyton floccosum* *Candida albicans* ATCC 90028 *Candida krusei* ATCC 6258 *Candida glabrata* HCCGL01 *Candida tropicalis* ATCC 750 *Candida parapsilosis* ATCC22019	500 μg/ml	Fungistatic toward *E. floccosum* Inactive against *T. rubrum, T. mentagrophytes*, *M. gypseum* and all tested *Candida* strains	de Morais et al. (2017)
Anti-viral	*E. montanum*	Leaves/polar fraction of an EtOH extract	*In vitro* Human immunodeficiency virus type-1 infected MT-4 cells		Inactive (EC_50_ > 166 μg/ml)	Cos et al. (2002a)
			*In vitro* *Herpes simplex* virus type 1 and semliki forest virus A7 Poliomyelitis virus type 1 strain 1A/S3 Coxsackie B2 virus Vesicular stomatitis virus T2 Measles Edmonston A	375 μg/ml 375 μg/ml 187.5 μg/ml 187.5 μg/ml 750 μg/ml	RF of 10^4^ RF of 10^3^ RF of 10^3^ RF of 10^2^ RF of 10^2^	Cos et al. (2002b)
Anti-diarrheal	*E. chinense*	Roots/EtOH extract and its chloroform fraction	*In vivo*: normal rats	Extract: 400 mg/kg p.o Fraction: 100 mg/kg p.o	↓ normal fecal excretion rate and water content	Prasad et al. (2013c)
			*In vivo*: castor oil-induced diarrhea rats		Delayed onset of diarrhea ↓ mean defecation, peristaltic index, intestinal fluid volume, prostaglandine E2-induced enteropooling Significant recovery of Na^+^ and K^+^ levels Prevention of alterations of nitric oxide, TBARS, SOD and catalase activities	
			*In vivo*: enteropathogenic *Escherichia coli*-induced diarrhea	Extract: 100 and 200 mg/kg p.o Fraction: 50 and 100 mg/kg p.o	↓ total number of stools, total number of diarrheal stools, weight of stools, mean defecation rate and water content ↑ Na^+^/K^+^-atpase activity, ↓intestinal secretion	Parmar et al. (2019a)
Anti-inflammatory	*E. campestre*	Roots/DCM-EtOH extract	*In vitro*: Peripheral blood mononuclear cells	6.25, 12.5 and 25 μg/ml	↓ T lymphocytes, including CD4^+^ and CD8^+^ cells ↓ IL-2 levels in the supernatant of the cell cultures	Santos et al. (2016)
	*E. cordatum*	Roots/petroleum ether, DCM, EtOH and H_2_O extracts	*In vitro*: Cyclooxygenase inhibition	250 μg/ml for organic extracts 2 mg/ml for the aqueous extract	Inhibition of COX-1 activity by 13–34% Inhibition of COX-2 activity by 6–35%	Ndhlala et al. (2011)
Anthelmintic	*E. griseum*	Leaves/EtOH extract	*In vitro*: *Echinostoma caproni*, *Schistosoma mansoni*, *Ancylostom aceylanicum*, *Heligmosomoides bakeri* and *Trichuris muris*		MLC of 20 μg/ml against third-stage larvae of *H. bakeri* MLC of 20 μg/ml toward newly transformed *S. mansoni schistosomula* MLC of 40 μg/ml against adult *S. mansoni* Moderate activity against third-stage larvae of *A. ceylanicum* no activity on adult *E. caproni* and *T. muris*	Koné et al. (2012)
			*In vivo*: mice harboring adult *S. mansoni*, *E. caproni* and *T. muris*	Single oral dose of 400 or 800 mg/kg 49 days post-infection	49.5% reduction of worm burden against adult *S. mansoni* at 400 mg/kg No activity against *E. caproni* and *T. muris* at 800 and 400 mg/kg, respectively	

a ↓: decrease or inhibition; ↑: increase; DPPH (1,1-di-phenyl-2-picrylhydrazyl), DCM: dichloromethane; PFRAP: potassium ferricyanide antioxidant power, IC_50_: concentration of a substance required for 50% of its maximal inhibitory effect, IZ: diameter of inhibition zone, MIC: minimum inhibitory concentration, MLC: minimum lethal concentration, p. o.: *per os*, RF: reduction factor of the viral titer, SOD: superoxide dismutase, TBARS: thiobarbituric acid reactive substances.

### Anticancer Activities

The *in vitro* antiproliferative or cytotoxic activity of extracts and compounds from *E. chinense*, *E. robustum* and *E. griseum* against various cancer cell lines has been reported suggesting some preliminary potential for cancer treatment. Using the MTT (3-(4,5-dimethylthiazolyl-2)-2,5-diphenyltetrazolium bromide) cell viability assay, a hexane root extract of *E. chinense* exhibited a moderate cytotoxic activity against human small-cell lung (NCI-H187) (IC_50_ of 12.0 μg/ml) and oral epidermal carcinoma (KB) (IC_50_ of 9.9 μg/ml) cells. Normal monkey kidney Vero cells were also moderately (IC_50_: 5.8 μg/ml) affected (Sutthivaiyakit et al., 2009). Khonklonginols A (**27**), F (**19**) and H (**38**), lupinifolinol (**23**), dehydrolupinifolinol (**18**) and flemichin D (**40**) from the roots of *E. chinense* exhibited a strong cytotoxicity (IC_50_ of 2.4–3.9 μg/ml) toward NCI-H187 cells while khonklonginol B (**28**), lupinifolin (**39**) and eriosemaone A (**34**) with IC_50_ values of 4.3, 6.5 and 6.0 μg/ml, respectively, were moderately cytotoxic (Sutthivaiyakit et al., 2009). Toward KB cells, compounds **23, 27**, **28**, **39** and **40** displayed a strong cytotoxic effect, while **18**, **19**, **34** and **38** showed a moderate effect. Compounds **18**, **19**, **27** and **38** displayed a significant cytotoxicity (IC_50_ of 6.4–11.1 μg/ml) toward Vero cells.

### Antioxidant Activities

By controlling the formation and scavenging of reactive oxygen and nitrogen species (ROS/RNS) as well as interrupting the related chain reactions and lipid peroxidation, antioxidants constitute the first line of defense against the genesis of various chronic and degenerative diseases. Different *in vitro* methodologies, including the DPPH (1,1-di-phenyl-2-picrylhydrazyl), nitric oxide radical and H_2_O_2_ scavenging assays, potassium ferricyanide antioxidant power (PFRAP), hydroxyl radical antioxidant capacity (HORAC), thiobarbituric acid reactive substances (TBARS) and the β-carotene bleaching test were used to evaluate the radical scavenging and antioxidant potential of members of the *Eriosema* genus.

Using a β-carotene bleaching assay, essential oil from fresh leaves of *E. englerianum* containing 3.65% ascaridole (**91**), 7.86% terpinolene (**92**) and 88.50% *O*-cymene (**93**) showed anti-oxidant activity with a color retention zone of 14.4 mm (Mmbengwa et al., 2009).

In the study by Prasad et al. (2013b), an ethanolic root extract of *E. chinense* (EEC) displayed a total antioxidant capacity of 90.2 μg/ml ascorbic acid equivalents. The PFRAP of the extract and the standard ascorbic acid was 0.26 and 0.42 μg/ml, respectively. The IC_50_ values of the extract for DPPH, hydrogen peroxide, nitric oxide and hydroxyl radical were 146.3, 221.0, 232.9 and 170.2 μg/ml, respectively, whereas those of the standards ascorbic acid (DPPH assay), rutin (H_2_O_2_ and nitric oxide) and butylated hydroxyanisole (hydroxyl) were 79.1, 82.9, 76.4 and 71.9 μg/ml, respectively. The antioxidant capacity of fractions (hexane, chloroform, ethyl acetate and aqueous) of EEC was also evaluated. A total antioxidant capacity of 76.0, 97.4, 89.5 and 44.6 μg/ml ascorbic acid equivalents, respectively, was determined. In contrast, very low DPPH (IC_50_ 163.1–312.1 μg/ml), nitric oxide (IC_50_ 201.8–372.2 μg/ml) and H_2_O_2_ (IC_50_ ≥ 187.7 μg/ml) radical scavenging activities as well as a low HORAC (IC_50_ ≥ 154.4 μg/ml) were observed for these fractions (Prasad et al., 2013a). Thongnest et al. (2013) evaluated the antioxidant activity of 11 flavonoids from the roots of *E. chinense* using a DPPH method. The most pronounced effects, close to the standard butylated hydroxytoluene (IC_50_ of 39 µM), were observed with compounds **10** (IC_50_ of 28 µM) and **20** (IC_50_ of 35 µM), while **11**, **12**, **23–25**, **27**, **33**, **40** and **49** with IC_50_ ≥ 252 μM, were inactive. In rats, a single oral pretreatment (30 min before castor oil challenge) with EEC (at 400 mg/kg), its chloroform fraction (at 100 mg/kg), and compounds **39** (10 mg/kg) and **81** (10 mg/kg) prevented the alterations of nitric oxide, TBARS (lipid peroxidation), superoxide dismutase and catalase activities in the castor oil-mediated diarrhea model (Prasad et al., 2013c; Prasad et al., 2017). Compound **81** (5 and 10 mg/kg) restored the oxidative status (nitric oxide level, SOD and CAT activities) in the enteropathogenic *Escherichia coli*-induced diarrhea rat model.

The anti-oxidant activity of an ethanol extract of twigs of *E. robustum*, its fractions and the isolated compounds **16** and **17**, **41** and **101** resulted in unphysiological IC_50_ values (≥1.13 mg/ml) using a DPPH method (Awouafack et al., 2013b). Using the same assay, compounds **5** and **72** displayed IC_50_ values of 340.3 and 524.6 μM, respectively, while that of ascorbic acid was 35.9 μM (Awouafack et al., 2018).

The antioxidant capacity of *Eriosema* species was evaluated *in vitro* by different methods addressing different mechanisms of action. Each of them has its strengths and weaknesses (Bibi Sadeer et al., 2020). The plant extracts and products are mixtures of compounds that can display their antioxidant activity through different mechanisms. Therefore, to better estimate the overall antioxidant activity of a sample, at least two (or even all) of these assays must be combined. Tan and Lim (2015) recommended a mix of single electron transfer based (DPPH, ABTS, FRAP, TBARS) and hydrogen atom transfer based (ORAC, HORAC, TRAP, crocin and β-carotene bleaching) assays. Among the six *in vitro* studies recorded in this review, just two (Prasad et al., 2013a; Prasad et al., 2013b) met with this requirement, while the other ones only used β-carotene bleaching (Mmbengwa et al., 2009) or DPPH (Awouafack et al., 2013b; Thongnest et al., 2013; Awouafack et al., 2018) assays. *In vitro* models are known to be not always reproducible in respective *in vivo* assays. Therefore, *in vivo* studies are necessary for the confirmation of such effects. Interestingly, the weakly active ethanol root extract of *E. chinense*, its chloroform fraction and the inactive compound **39** using DPPH method, restored the alterations of nitric oxide, TBARS, superoxide dismutase and catalase activities in the castor oil-mediated diarrhea model in rats (Prasad et al., 2013c). Although data at this level seems to be naïve and preliminary for any conclusion, they are indicator of the antioxidant potential of members of the *Eriosema* genus. However, more species need to be investigated *in vitro and in vivo*.

### Estrogenic and Aryl Hydrocarbon Agonistic Properties

Estrogens deficiency (natural or surgical) is associated with numerous health problems including urogenital atrophy, hot flushes, osteoporosis and increased risk of cardiovascular diseases (Calleja-Agius and Brincat, 2015; Nappi and Cucinella, 2020). Given the adverse outcomes associated with the long-term usage of the hormone replacement therapy e.g. increased risk of endometrial and breast cancer, stroke and pulmonary thromboembolism, phytoestrogens have gained and continue to receive great interest.

The estrogenic properties of *E. laurentii*, traditionally used against female infertility, gynecological and menopausal complaints have been evaluated *in vitro* and *in vivo*. *In vitro,* a methanol extract of the aerial parts (AEL), its fractions and the predominant secondary metabolites **43**, **44** and **73** induced a significant and concentration-dependent β-galactosidase activity in the ERα yeast two-hybrid assay (yERα) (Ateba et al., 2013; Ateba et al., 2014b). At 25 μg/ml, AEL was as potent as 10 nM estradiol (Ateba et al., 2013). Compounds **43**, **44** and **73** displayed EC_50_ values of 0.32 µM, 6.1 µM and 21.4 nM, respectively, while that of 17β-estradiol (E_2_) was 0.27 nM. All the three compounds were full ERα agonists as they displayed the maximal efficacy of E_2_ although at much higher concentrations (Ateba et al., 2014b). A methanolic extract of the underground parts of *E. laurentii* (UEL) was also highly active in yERα (Ateba et al., 2016b). The concentration of 10 μg/ml was as potent as 0.5 nM E_2_. However, compounds **22**, **62** and **63** from this extract did not show any yERα activity (Ateba et al., 2016b).

*In vivo*, a 3-days uterotrophic assay and a 9-weeks oral treatment in ovariectomized adult rats were tested. AEL at the doses of 50, 100 and 200 mg/kg *p. o*. did not induce endometrium proliferation either in the 3-days or the 9-weeks treatment regimens, but induced vaginal stratification and cornification (Ateba et al., 2013). This tissue-dependent effect suggests that AEL could prevent vaginal dryness experienced by menopausal women, while not inducing the unwanted uterine proliferation. In the other hand, the decoction of aerial parts of *E. laurentii* had no significant effect on the uterine and vaginal endpoints at all tested doses following a 3-days uterotrophic assay (Ateba et al., 2014c).

Studies reported the repressive effects of aryl hydrocarbon receptor (AhR) agonists on ERα estrogen-sensitive cancer (endometrium and breast) cells (Tsuchiya et al., 2005; Okino et al., 2009; Marconett et al., 2010; Labrecque et al., 2012). In line with this, the agonistic properties of AEL and compounds were evaluated. AEL exhibited a dose-dependent and significant AhR β-galactosidase activity from 5 μg/ml. The activity at 83 μg/ml was similar to that of 50 nM β-naphthoflavone (Ateba et al., 2013). For the first time, **44** (EC_50_ not determined) and **73** (EC_50_ of 1.34 µM) were found as partial agonists of AhR, whereas **43** was not active (Ateba et al., 2014b).

Classical, suitable and reproducible systems/models have been used to evaluate estrogenic and aryl hydrocarbon agonistic properties of *E. laurentii in vitro* (Jungbauer and Beck, 2002; Reiter et al., 2009) and *in vivo* (OECD, 2007; Mvondo et al., 2011). Accordingly, all data reported in this section demonstrated the potential of *E. laurentii* for managing various gynecological and menopausal complaints. However, this species is also traditionally used for treating female infertility. Studies in that domain are needed. Similarly, a wide traditional use of *E. cordatum* in South Africa against female infertility has been recorded. Nevertheless, no published study demonstrates this potential till now.

### Antiosteoporosis Activity

Osteoporosis is a skeletal disorder characterized by a deterioration of bone tissue and disruption of bone microarchitecture that lead to low bone mass and strength, predisposing to an increased risk of fracture (Pisani et al., 2016). It affects both sexes and is mainly linked with the age-related decrease of estradiol and testosterone. Therapeutic agents that would not only inhibit bone resorption but also simultaneously stimulate bone formation would be most favorable (Abdelsamie et al., 2019).

Using a well-known postmenopausal-like model of osteoporosis (ovariectomy) in rats, Ateba et al. (2013) evaluated the effects of a 9-weeks oral treatment with a methanolic extract of the aerial parts of *E. laurentii*. The extract significantly increased the dry femur weight (at 50 and 200 mg/kg), and calcium levels in serum (at 50 and 200 mg/kg) and femur (at all tested doses) compared with ovariectomized animals. The serum levels of inorganic phosphorus decreased, while the femur levels increased in a dose-dependent manner. Although the dose of 200 mg/kg was not able to completely reverse the ovariectomy-increased alkaline phosphatase (a biomarker for osteoblastic activity and bone remodeling), it decreased it by ∼15%. Based on the increased in femur mass, and femur calcium and inorganic phosphorus contents, the authors concluded that bone formation exceeded resorption suggesting the inhibition of bone resorption and activation of bone formation.

There is growing interest in 17*β*-hydroxysteroid dehydrogenase type 2 (17*β*-HSD2), an enzyme able to regulate the intracellular concentration of estradiol and testosterone by transforming them into the less potent forms estrone and androstenedione (Marchais-Oberwinkler et al., 2011) in tissues where it is expressed, such as bone (Dong et al., 1998; Eyre et al., 1998). Blockade of this enzyme is thought to increase intracellular estradiol and testosterone, which thereby inhibits bone resorption by osteoclasts and stimulates bone formation by osteoblasts (Abdelsamie et al., 2019). In line with this 17*β*-HSD2 is more and more considered as target for antiosteoporosis treatment. In an *in vitro* assay using lysates of cells expressing the recombinant human enzyme 17*β*-HSD2, Vuorinen et al. (2017) tested 36 hit molecules, including **44** and **73** from *E. laurentii*. These compounds with respective IC_50_ values of 1.52 and 2.03 µM were among the most active 17*β*-HSD2 inhibitors tested. However, they selectively inhibited 17*β*-HSD1 (that catalyzes the reverse reaction) over 17*β*-HSD2 and therefore cannot be suitable lead structures for a possible therapeutic use (Vuorinen et al., 2017).

### Erectile Dysfunction and Impotence

Erectile dysfunction (ED) as well as impotence is a multifactorial (psychogenic, neurogenic, vasculogenic, endocrine/metabolic, end organ disease and iatrogenic) health issue common in 20–30% of adult men worldwide (McMahon, 2019; Taylor et al., 2019). It negatively impacts patient’s sexual satisfaction and psychological well-being, leading to a greater prevalence of anxiety and depression. Lifestyle changes in combination with oral phosphodiesterase type 5 inhibitors (PDE5Is: sildenafil, tadalafil, vardenafil and avanafil) are typically first-line treatments of most etiologies of ED (Hatzimouratidis et al., 2016; Krzastek et al., 2019; Moses et al., 2019). PDE5Is increase arterial blood flow into the penis (via relaxion of the *corpus cavernosum* and deep penile artery smooth muscles) and reduce the venous outflow leading to the penile erection. Among PED5Is, only sildenafil is cost-effective as it is the only PDE5-I with a generic option. PED5Is are commonly associated with side effects (flushing, hypotension, headache, dyspepsia, back pain, myalgia, dizziness, blurred vision and rhinitis) and a lot of contraindications that contribute to significant treatment dropout rates (Yuan et al., 2013; Yafi et al., 2018). Moreover, up to 40% of ED patients fail to respond sufficiently to the maximum dose of PDE5Is (Porst et al., 2013).

From ancient times, plants have served as a dependable source of medicines for treating ED. In the *Eriosema* genus, *E. cordatum, E. kraussianum* and *E. salignum* are traditionally or locally used as aphrodisiac or against male sexual disorders (ED, impotence). However, only compounds from *E. kraussianum* were evaluated until now. In 2002, Drewes et al. investigated the capacity of pyrano-isoflavones **64–68** to relax the *corpus cavernosum* of rabbits. Compounds **64** and **65** displayed a dose-dependent activity, while **66–68** were inactive. At the low concentration of 78 ng/ml, **64** and **65** displayed 85 and 65% of the relaxation found with sildenafil citrate, respectively. Rather to induce relaxation, **66** and **68** caused contraction of *corpus cavernosum* tissue (Drewes et al., 2004). Accordingly, the authors indicated that the overall effect of the extract as used in traditional medicine is mainly determined by the activities of **64** and **65**, present as the major constituents.

*Eriosema cordatum, E. kraussianum* and *E. salignum* and the other *Eriosema* species that come under the *isiZulu* indigenous umbrella name of “uBangalala” are widely traditionally used mainly in South Africa for curing or alleviating ED and/or impotence (Bryant, 1966; Hutchings et al., 1996). However, despite their high reputation in that domain, the investigation only focused on *E. kraussianum* with two papers published more than 15 years ago. In line with this, these *Eriosema* species traditionally used against ED remain underexplored and even seem to be abandoned. Given the burden of this sensitive topic, they require more research attention.

### Hypoglycemic and Anti-diabetic Effects

Diabetes is a major global health threat. This metabolic disorder is caused by a lack (absolute or relative) of insulin and/or resistance to insulin that lead to chronic hyperglycemia.

ED is a common comorbidity of diabetes mellitus. It can be caused by impaired hemodynamic mechanisms in the penile and ischemic vasculature that occur in diabetes mellitus (Rendell et al., 1999). There is a consensus that lifestyle changes and risk factor modification must precede or accompany any pharmacological or psychological ED treatment (Hatzimouratidis et al., 2016; Hackett et al., 2018). In other words, the amelioration of diabetes risk factors may improve erectile function. Acute oral treatment with a *E. kraussianum* rootstock hydro-alcoholic extract (40–320 mg/kg) dose-dependently and significantly reduced fasting blood glucose levels in normoglycemic and streptozotocin-treated diabetic rats from 2 to 8 h after treatment with peak effects after 4 h (Ojewole et al., 2007). Using an oral glucose tolerance test, a pretreatment with the extract 20 min before challenge significantly reduced the peak blood glucose levels. Similar effects were obtained with 80 mg/kg *p. o.* of kraussianones 1 and 2 in normoglycemic rats (Ojewole et al., 2006).

In alloxan-induced diabetic Wistar rats, both aqueous and ethanolic leaf extracts of *E. psoraleoides* (at 200 and 400 mg/kg) significantly decreased fasting blood glucose levels from day 6 of a 7-days treatment (Nduka et al., 2018; Nduka et al., 2019). Using the same model, Elechi et al. (2019) investigated the effects of *n*-hexane, *n*-hexane/dichloromethane (1:1 v/v), dichloromethane, and dichloromethane/methanol (19:1 v/v) fractions of the *n*-hexane leaf extract of *E. psoraleoides* on the fasting blood glucose levels. The *n*-hexane/dichloromethane and the dichloromethane fractions at the dose of 200 mg/kg produced a significant decrease in fasting blood glucose levels from day 2–7, the effects of the dichloromethane fraction being higher or very close to that of 4 mg/kg glibenclamide (Elechi et al., 2019).

*Eriosema glomeratum* claimed to be traditionally used against diabetes (Lawin et al., 2015) is not yet investigated in relation to the metabolic disorder.

### Cardiovascular protection: dyslipidemia, vasodilatation and preeclampsia

Cardiovascular disease remains the leading cause of death worldwide (Global Burden of Disease Study 2017; Causes of Death Collaborators, 2018). Managing its modifiable risk factors such as hypertension and dyslipidemia constitutes key targets of public health organizations over the world (Berman et al., 2019; Michos et al., 2019).

Increased risk of cardiovascular diseases such dyslipidemia is well known to be associated with menopause. Using a postmenopause-like model (ovariectomized Wistar rats), a 9-weeks oral treatment with a methanol extract of the aerial parts of *E. laurentii* significantly decreased the total cholesterol (TC) and LDL levels at 200 mg/kg. Increased HDL as well as a non-significant decrease of triglycerides (TG) levels were also observed at all tested doses (50, 100 and 200 mg/kg). The extract decreased the ovariectomy-increased TC/HDL and LDL/HDL ratios. A significant decrease of the atherogenic plasma index [AIP: log_10_ (TG/HDL)] was determined at all tested doses (Ateba et al., 2013). Individual cholesterol risk factors are not sufficient to assess cardiovascular risk (Michos et al., 2019). A huge body of evidence supports AIP as one of the strongest predictive indicator of cardiovascular disease risk (Dobiášová et al., 2004; Edwards et al., 2017) also in postmenopausal women (Wu et al., 2018; Barua et al., 2019). This parameter has been evaluated in the study by Ateba et al. (2013) indicating the potential of *E. laurentii* in the management of dyslipidemia in postmenopausal women.

Ojewole et al. (2006) investigated the vasodilatory effects of **64** and **65** (100–2,000 μg/ml) from the rootstock of *E. kraussianum* in isolated portal veins. Both constituents, after provoked initial slight contraction, induced a dose-dependent secondary and pronounced vasorelaxant effect, probably by affecting calcium mobilization and/or sequestration, and possibly also, calcium release from its various tissue stores.

Preeclampsia is the most common pregnancy-related complication worldwide, affecting 5–7% of all pregnancies with estimated 70,000 maternal deaths and 500,000 fetal and neonatal deaths each year (Rana et al., 2019). This incidence probably remains underestimated due to underreporting (Mayrink et al., 2018). The options for prevention and treatment of this hypertensive disorder are extremely limited, especially in resource-limited settings (Lemoine and Thadhani, 2019) where almost all of the deaths occur (Oyston and Baker, 2020). In this context, medicinal plants and herbs are an opportunity. In preeclampsia, the abnormal placentation characterized by impaired spiral artery remodeling lead to an ischemic placenta, which releases factors such as anti-angiogenic factors sFlt-1 (soluble fms-like tyrosine kinase-1) and sEng (soluble Endoglin), responsible for vascular dysfunction, into the maternal circulation (Chang et al., 2018; Eddy et al., 2018; Jena et al., 2020). Based on the vasodilatory properties of **65** (Drewes et al., 2002; Ojewole et al., 2006), Ramesar et al. (2012) investigated its effects in the l-NAME-induced preeclamptic Sprague-Dawley rat model. Subcutaneously administered at 10 mg/kg for 12 consecutive days, the compound decreased fetal mortality, demonstrated a trend toward increasing birth and placental weights by improving placental perfusion, reducing blood pressure amplification through a NO-independent mechanism, and decreasing the plasma levels of sFlt-1and sEng.

Globally, based on the *in vivo* models used and the results obtained, the investigated *Eriosema* species display a potential in the management of modifiable risk factors (hypertension, dyslipidemia) of the cardiovascular disease.

### Acetylcholinesterase Inhibition

The inflammation-associated cognitive decline is mainly due to degeneration of central cholinergic neurons or cholinergic impairment, making acetylcholinesterase inhibitors the main class of drugs currently used in the treatment of mild cognitive impairment and Alzheimer’s disease (Benfante et al., 2019). In a study by Ndhlala et al. (2011), an aqueous extract of the roots of *E. cordatum* with an IC_50_ value of 756.6 μg/ml showed a very low acetyl cholinesterase inhibitory activity.

### Anti-Microbial Activity

Infectious diseases are among the most important cause of morbidity and mortality worldwide. This is worsened by drug resistance, one of the greatest challenges of the 21st century (Hofer, 2019; Tacconelli and Pezzani, 2019). The importance of plants as a source of effective anti-microbial agents is well established. Several reports dealing with the anti-bacterial and anti-fungal effects of *Eriosema* species have been documented.

Flavonoids from the roots of *E. chinense* were tested against Gram-negative (*Escherichia coli* 25,922*, Klebsiella pneumoniae* and *Pseudomonas aeruginosa*) and Gram-positive (*Bacillus cereus, Enterococcus faecalis, Staphylococcus aureus,* methicillin-resistant *S. aureus* (*S. aureus* MRSA), *Staphylococcus epidermidis, Streptococcus agalactiae, Streptococcus pyogenes* and *Listeria monocytogenes*) bacteria, and *Candida albicans* using agar dilution technique. Compound **27** displayed a strong activity (minimum inhibitory concentration (MIC) of 2.3 μg/ml) against *S. agalactiae* and *S. pyogenes*. MIC values of 2.3 μg/ml (toward *S. pyogenes*), 4.7 μg/ml (toward *B. cereus* and *S. agalactiae*) and 9.4 μg/ml (toward *L. monocytogenes*, *S. aureus* MRSA, *S. epidermidis*) were observed with compound **23**. Compound **40** displayed MIC values of 4.7 μg/ml toward *B. cereus, E. faecalis, L. monocytogenes*, and all *Streptococcus* and *Staphylococcus* strains. Compound **25** significantly inhibited the growth of *B. cereus* (MIC of 2.3 μg/ml)*, S. pyogenes* (MIC of 2.3 μg/ml), *S. aureus* (MIC of 4.7 μg/ml)*, S. aureus* MRSA (MIC of 4.7 μg/ml), *S. agalactiae* (MIC of 4.7 μg/ml), and *E. faecalis* (MIC of 9.4 μg/ml) and *L. monocytogenes* (MIC of 9.4 μg/ml). With **20**, a MIC value of 9.4 μg/ml was obtained against *B. cereus*, *S. aureus, S. aureus* MRSA, *S. agalactiae, S. epidermidis* and *S. pyogenes*, and of 18.8 μg/ml toward *E. faecalis* and *L. monocytogenes*. The standard amphotericin B resulted in a MIC of 0.12 μg/ml against *C. albicans*, while chloramphenicol displayed MIC values of 10 μg/ml against *E. coli* 25,922, *K. pneumoniae*, *B. cereus, E. faecalis*, *L. monocytogenes, S. aureus* and *S. aureus* MRSA, and of 1 μg/ml toward *S. agalactiae, S. epidermidis* and *S. pyogenes* (Thongnest et al., 2013).

Dichloromethane and ethanolic root extracts of *E. cordatum* exhibited moderate antibacterial activities against *S. aureus*, *B. subtilis*, *E. coli* and *K. pneumoniae* (Ndhlala et al., 2011). The ethanol extract showed MIC values of 390 μg/ml toward all these microbial species, while that of the dichloromethane extract were 195 μg/ml against *S. aureus* and *B. subtilis*, and 390 μg/ml against *E. coli* and *K. pneumoniae*. Petroleum ether and water root extracts were not active (MIC ≥1.56 mg/ml) against all the tested microbial species. The MIC of the standard neomycin ranged between 0.8 μg/ml and 1.6 μg/ml (Ndhlala et al., 2011).

The ethanolic root extract of *E. chinense* (Prasad et al., 2013c) as well as its hexane, chloroform and ethyl acetate fractions (Prasad et al., 2013b) displayed MIC >195 μg/ml against reference bacterial strains *E. coli* ATCC25922, *Shigella flexneri* ATCC12022, *P. aeruginosa* ATCC27893, *S. aureus* ATCC25323 and clinical isolates of *Salmonella typhi*, *Shigella dysenteriae*, *Proteus vulgaris*, *K. pneumoniae*, *Shigella boydii*, *B. cereus* and *E. faecalis*. In these studies, IZ values (24.1–30.7 mm) of the standard ciprofloxacin (0.5 mg/ml) were determined, but MIC values not stated. Compounds **18**, **34**, **39** and **40** from the roots of *E. chinense* showed MIC values of 12.5 μg/ml against *Mycobacterium tuberculosis* H37Ra, while that of **23**, **27** and **38** were 25 μg/ml. Those of the standards isoniazid and streptomycin were 0.023–0.046 and 0.156–0.313 μg/ml, respectively (Sutthivaiyakit et al., 2009).

Using the disk diffusion method, the essential oil from the fresh leaves of *E. englerianum* (1, 2, 5 and 10 μL/ml) was tested against eight different bacteria (*S. aureus*, *E. coli*, *P. aeruginosa*, *Bacillus subtilis*, *K. pneumoniae*, *P. vulgaris*, *Clostridium sporogenes* and *Acinetobacter calcoaceticus*). Considered as positive result by authors, a diameter of inhibition zone (IZ) ≥ 7 mm was observed at 5 μL/ml against *A. calcoaceticus*, and at 10 μL/ml against *C. sporogenes* and *A. calcoaceticus* (Mmbengwa et al., 2009). Against opportunistic fungi, a concentration-dependent inhibition of *C. albicans* (24.2–42.6%), *Aspergillus niger* (34.7–49.7%) and *Aspergillus flavus* (27.5–39.3%) at concentrations of 1–10 μL/ml was seen, while nystatin inhibited them by 84.3, 76.3 and 76.3%, respectively. However, the concentration of nystatin displaying this activity was not indicated (Mmbengwa et al., 2009).

Through the broth dilution method, methanol/dichloromethane (1:1, v/v) extracts of stems and leaves of *E. glomeratum* showed MIC values ranging between 50 and 1000 μg/ml against *S. aureus*, *K. pneumoniae*, *Morexella catarrhalis*, *Mycobacterium smegmatis* and *Mycobacterium aurum* (Fomogne-Fodjoet al., 2014). The stem extract displayed the highest activity with MIC values of 65 and 50 μg/ml against *M. smegmatis* and *M. aurum,* respectively, whereas the leaf extract was most active against *M. smegmatis* (MIC 130 μg/ml). Against these two *Mycobacterium* species, the standard ciprofloxacin displayed MIC values of 78 and 1.5 ng/ml, respectively. Compounds **1**, **2**, **13**, **47** and **85** from the methanol/dichloromethane (1:1 v/v) extract of the whole plant of *E. glomeratum* were tested at a concentration of 1 μg/ml against bacteria (*Bacillus megaterium*, *E. coli*), the green alga *Chlorella fusca* and the fungus *Microbotryum violaceum* using an agar diffusion assay (Awouafack et al., 2008). Compound **1** displayed IZ of 7 mm toward *B. megaterium*, *C. fusca* and *M. violaceum*, and of 10 mm against *E. coli*. IZ values of 10, 8, 9 and 13 mm were observed with **2** against *B. megaterium*, *E. coli*, *C. fusca* and *M. violaceum*, respectively. Compounds **13** and **47** were only active (IZ of 10 mm) against *C. fusca*, while **85** was inactive against all four microbial species. In this study, the standards were also tested at a concentration of 1 μg/ml. Tetracycline displayed IZ of 18 mm against bacteria, while an IZ of 20 mm was observed with nystatin against *M. violaceum.* The IZ of the standard actidione was 35 and 50 mm against the alga and fungus, respectively (Awouafack et al., 2008).

A polar fraction of an ethanolic leaf extract of *E. montanum* displayed a low antimicrobial activity with a MIC > 750 μg/ml against bacteria (*B. cereus* ATCC 14579, *Enterobacter cloacae* ATCC 13047, *E. coli* ATCC 8739, *K. pneumoniae* ATCC 13883, *Mycobacterium fortuitum* ATCC 6841, *P. vulgaris* ATCC 13315, *P. aeruginosa* ATCC 15442, *Salmonella typhimurium* ATCC 13311, *S. aureus* ATCC 6538, *Streptococcus pyogenes* ATCC 12344) and fungi (*C. albicans* ATCC 10231, isolates of *Trichophyton rubrum* RV 58125, *Epidermophyton floccosum* RV 71625 and *Microsporum canis* RV 66973) (Cos et al., 2002b).

Methanolic extracts of the bark and wood of twigs of *E. psoraleoides* displayed very high MIC values (≥1.25 mg/ml) against *Streptococcus mutans* HG 982, *Actinomyces viscosus* HG485 and *C. albicans* HG392 in the agar dilution method (Khan et al., 2000). MIC values of 8, 32 and 128 μg/ml against these microorganisms were observed with the standard chlorhexidine, respectively. Using a bioautography agar overlay method, Runyoro et al. (2006) reported that a methanolic extract of the stem bark of *E. psoraleoides* was not active against *C. albicans*. A methanolic leaf extract as well as its *n*-hexane and ethyl acetate fractions were tested against *E. coli* ATCC35219, *S. aureus* ATCC25923 and clinical isolates of *S. aureus*, *P. vulgaris*, *Klebsiella aerogenes*, *P. aeruginosa*, and *E. coli* and displayed MIC >1.26 mg/ml, except the *n*-hexane fraction with a MIC value of 150 μg/ml toward *S. aureus* ATCC. The MIC value of the standard gentamicin was not reported (Elechi and Igboh, 2017).

An ethanolic extract from the twigs of *E. robustum* showed a MIC value of 80 μg/ml against *S. aureus*, *E. faecalis* and *E. coli* and of 310 μg/ml toward *P. aeruginosa*. Significant differences in the activity were observed against the fungi *Aspergillus fumigatus* (MIC 160 μg/ml), *Cryptococcus neoformans* (MIC 630 μg/ml), *C. albicans* and *C. albicans* ATCC (MIC 1,250 μg/ml) (Awouafack et al., 2013a). Compounds from this extract showed either moderate or low activity against these microbial species. Moderate antimicrobial activities **(**MIC 63 or 65 μg/ml) were observed with **16** and **17** against *C. albicans* ATCC, *A. fumigatus* and *P. aeruginosa*. **17** also showed a moderate activity against *Cryptococcus neoformans* and *E. faecalis*. A MIC of 63 or 65 μg/ml was observed with **107** against *P. aeruginosa* and with **87** against *A. fumigatus* and *P. aeruginosa*; **101** against *C. albicans* ATCC and *P. eruginosa*; **99** against *S. aureus* and *P. aeruginosa*; **88** against *A. fumigatus*, *C. neoformans*, *P. aeruginosa* and *E. faecalis*; **41** against *C. albicans* ATCC, *S. aureus*, *P. aeruginosa*, *E. faecalis* and *E. coli* (Awouafack et al., 2013a). The standard gentamicin showed a MIC <3.91 μg/ml against bacteria, while that of amphotericin B was 30 μg/ml toward *C. albicans* (isolate) and *C. albicans* (ATCC), >250 μg/ml against *C. neoformans* and 125 μg/ml against *A. fumigatus*. Using a microdilution method, **5** and **72** from the ethanol extract of twigs of *E. robustum* displayed weak antimicrobial activity (MICs >150 μg/ml) against *Bacillus subtilis*, *S. aureus*, *K. pneumoniae*, *E. coli*, *C. albicans*, and *Saccharomyces cerevisiae* (Awouafack et al., 2018) In this study, the tested concentrations or MIC values of standard antibiotics ampicillin, kanamycin, and cycloheximide were not indicated.

Using the broth microdilution method, the antifungal activity of methanolic leaf extracts (500 μg/ml) of *Eriosema campestre* var. *campestre*, *Eriosema campestre* var. *macrophyllum*, *Eriosema glabrum* Mart. ex Benth., *Eriosema heterophyllum* Benth., *Eriosema longifolium* Benth. and *Eriosema tacuaremboense* Arechav. was evaluated. After 72 h incubation, *E. heterophyllum* was fungistatic against *T. rubrum*, *Trichophyton mentagrophytes* and *Microsporum gypseum* and fungicidal against *Epidermophyton floccosum*. The extracts of *E. campestre* var. *macrophyllum* and *E. glabrum,* respectively, displayed fungistatic and fungicidal activities against *T. mentagrophytes* and *E. floccosum*. The extract of *E. campestre* var. *campestre* was fungistatic toward *T. rubrum*, *T. mentagrophytes* and *E. floccosum*. Moreover, all these *Eriosema* species were not active against *C. albicans*, *Candida krusei*, *Candida glabrata*, *Candida tropicalis* and *Candida parapsilosis* after 48 h incubation (de Morais et al., 2017).

The bioassay-guided fractionation of a dichloromethane root extract of *E. tuberosum* using a TLC bioautography assay led to the isolation of compounds **34–36**, **40**, **62** and **75**. Amounts of 5 µg of **35**, **36**, **40** and **62**, and 10 µg of **34** on TLC plates prevented the growth of *Cladosporium cucumerinum*, while 1 µg of **34**, **36**, **40** and **62**, and 5 µg of **35** inhibited the growth of *Candida albicans*. Eriosematin (**75**) was not active against *C. cucumerinum* and *C. albicans* (Ma et al., 1995). **77** from the same extract inhibited the growth of both fungi at 2.5 µg, while **76**, **78** and **79** were not active (Ma et al., 1996a). Compounds **80** and **81** exhibited a strong activity (inhibition at 0.5 µg) against *C. cucumerinum* and a weak activity (inhibition at 30 µg) against *C. albicans* (Ma et al., 1996b). Moreover, 10 and 30 µg of **39** were active against *C. cucumerinum* and *C. albicans*, respectively. In the same assay, 2 µg of **98** and 3µg, each, of **94**, **96** and **104** inhibited the growth of *C. cucumerinum*, **82**, **105** and **106** were not active against *C. cucumerinum* (Ma et al., 1999).

In a study by Sianglum et al. (2019), lupinifolin (**39**) demonstrated an antimicrobial activity against *E. faecalis* ATCC29212, *S. aureus* ATCC25923 as well as susceptible and multidrug-resistant enterococcal clinical isolates (*E. faecalis* and *E. faecium*) in the standard broth microdilution method. Although the tested compound was not extracted from an *Eriosema* species, it has been reported in roots of *E. chinense* and *E. robustum.* In that study, **39** displayed activity against all susceptible and resistant strains with MICs and MBCs (minimum bactericidal concentration) ranging between 0.5 and 2 μg/ml and between 2 and 16 μg/ml, respectively. Toward all the strains, compound **39** with MIC values of 0.5–2 μg/ml was more active than vancomycin (MIC of 1–256 μg/ml). It increased membrane permeability and caused loss of salt tolerance. On *E. faecalis* ATCC29212, *E. faecalis* HTY0037 (a multidrug resistance isolate) and *E. faecium* HTY0256 (a strong biofilm-producing vancomycin-resistant enterococci isolate), after 2 h incubation it displayed significant rapid antibacterial activity. Moreover, an antibiofilm-producing activity of **39** against four MDR enterococci was shown.

Eighteen articles dealing with the antimicrobial activity of *Eriosema* species have been published using disk diffusion, broth dilution and thin layer chromatography (TLC) bioautography methods. The “traditional” inhibitory zone (IZ) determined by the disc diffusion method depends on the coefficient of diffusion of compounds, highly polar substances diffusing more easily in agar and displaying a high IZ and *vice versa* (Tan and Lim, 2015). Therefore, this assay is only useful for a simple qualitative screening as it does not allow the determination of the real amount of the antimicrobial agent that diffuses into the agar, impeding the determination of MICs and MBCs (Balouiri et al., 2016). Based on the MIC values, extracts (or compounds) display: *i*) a strong antimicrobial activity *in vitro* if the MIC ≤100 μg/ml (or 10 μg/ml), *ii*) moderate if 100 < MIC ≤625 μg/ml (or 10 < MIC ≤100 μg/ml), *iii*) and low if MIC > 625 μg/ml (or >100 μg/ml) (Eloff, 2004; Ríos and Recio, 2005; Kuete, 2010). Based on this classification, several extracts and compounds from *Eriosema* species displayed promising antimicrobial activity. Unfortunately, in several studies positive controls were not included or their concentration was not stated. Although some studies are suggesting the potential of few compounds to act as an antimicrobial drug against several microorganisms, these deficiencies weaken the evidence.

### Anti-Viral Activity

Cos et al. (2002a) assessed the anti-human immunodeficiency virus type-1 (HIV-1) effects of a polar fraction of an ethanolic leaf extract of *Eriosema montanum* Baker f. using a tetrazolium-based colorimetric assay in infected MT-4 cells. The polar fraction was obtained suspending the extract in 60% methanol and defatting with petroleum ether. The polar fraction with an EC_50_ value >166 μg/ml did not protect the HIV-infected cells.

At the maximal non-toxic concentrations of 375 μg/ml, this polar fraction exhibited a pronounced antiviral activity (reduction factor of the viral titer (RF) of 10^3^ – 10^4^) against Herpes simplex virus type 1, Poliomyelitis virus type 1 strain 1A/S3 and Semliki forest virus A7, while at 187.5 μg/ml it displayed a RF of 10^3^ against Coxsackie B2 virus. RF values of 10^2^ were observed against Vesicular stomatitis virus T2 and measles Edmonston A at 187.5 and 750 μg/ml, respectively (Cos et al., 2002b).

### Anti-Diarrheal Activity

Diarrheal diseases are a leading cause of mortality and morbidity worldwide, mainly among children under-five in low developing countries. Defined as the discharge of 4 or more semisolid or watery feces per day, diarrhea involves an increase in intestinal fluid volume, in the frequency of bowel movement, wet stool and abdominal cramps, leading to loss of electrolytes and water. Its treatment and management depend on the duration and specific etiology which can be infectious or not. Over the world, many people or communities still use traditional herbs to treat a variety of diseases including diarrhea. As depicted in Table 1, several *Eriosema* species are used traditionally for diarrhea.

Castor oil-induced diarrhea is known as an efficient model for the initial screening for antimotility, antisecretory and anti-inflammatory compounds. In the gut, ricinoleic acid (castor oil metabolite) causes irritation and inflammation in bowels leading to diarrhea (stomach cramp, increased peristaltic activity and intestinal fluid volume) (Matias et al., 1978; Racusen and Binder, 1979). An ethanol root extract of *E. chinense* (EEC; at 400 mg/kg), its chloroform fraction (at 100 mg/kg; CEC), and compounds **39** and **81** (at 10 mg/kg) reduced the normal fecal excretion rate and water content 3, 5 and 7 h after the oral treatment of rats. In castor oil-induced diarrhea, these treatments delayed the onset of diarrhea and decreased the mean defecation in a dose-dependent manner. At the same dosages, the extract, the fraction and **39** and **81** significantly reduced the peristaltic index, the intestinal fluid volume and PGE2-induced enteropooling, and demonstrated a significant recovery from intestinal fluid loss of Na^+^ and K^+^ (Prasad et al., 2013c; Prasad et al., 2017).

Enteropathogenic *E. coli* (EPEC) is a common cause of moderate to severe water diarrhea in children, accompanied with fever, vomiting and a high hazard of death. In an enteropathogenic *E. coli*-induced diarrhea rat model, EEC (100 and 200 mg/kg), CEC (50 and 100 mg/kg) and **81** (5 and 10 mg/kg) induced a significant recovery from diarrhea characterized by the reduction in total number of stools, total number of diarrheal stools, weight of stools, mean defecation rate and water content of stools 6 h after induction of diarrhea (Parmar et al., 2019a; Parmar et al., 2019b). At the higher tested doses, EEC, CEC and eriosematin E displayed diarrhea scores very close to that of 5.7 mg/kg norfloxacin. They also increased Na^+^/K^+^-ATPase activity to a higher level than that observed in the normal rats, resulting in reduced intestinal secretion.

Both infectious and non-infectious (chemically induced) reliable models were used to evaluate the anti-diarrheal potential of *Eriosema* species *in vivo*. However, from five species traditionally claimed to be anti-diarrheal (*E. affine*, *E. chinense*, *E. griseum*, *E. psoraleoides* and *E. tuberosum*), only the ethanolic root extract of *E. chinense* and compounds **39** and **81** were investigated, indicating that in the *Eriosema* genus this domain is underexplored. Given the promising results obtained with one extract, it would be interesting to extend the exploration to the other *Eriosema* species traditionally used against diarrhea.

### Anti-Inflammatory Activity

Intestinal disorders often co-occur with inflammation and dysmotility. In enteropathogenic *E. coli-*induced diarrhea, 10 mg/kg of **81** from roots of *E. chinense* for 24 h significantly decreased the expression of pro-inflammatory cytokines IL-1β and TNF-α in colonic tissues (Parmar et al., 2019b).

Using peripheral blood mononuclear cells, Santos et al. (2016) investigated the impact of a dichloromethane-ethanol extract of *E. campestre* (6.25, 12.5 and 25 μg/ml) on pathological processes of chronic inflammatory diseases. In a concentration-dependent manner, the extract inhibited the proliferation of T lymphocytes, including CD4^+^ and CD8^+^ cells, and decreased IL-2 levels in the supernatant of the cell cultures, a cytokine essential for the expansion of T lymphocytes.

Cyclooxygenase (COX) inhibitors are known to be a therapeutic target in inflammatory and neuroinflammatory diseases (Grosser et al., 2017; Dhir, 2019). The COX-1 and COX-2 inhibitory activities of extracts of the roots of *E. cordatum* were investigated by Ndhlala et al. (2011). At 250 μg/ml, petroleum ether, dichloromethane and ethanolic extracts weakly inhibited COX-1 activity by 13, 25 and 34%, respectively. A weak inhibition of COX-2 by 6, 35 and 10%, respectively, with 250 μg/ml of the petroleum ether, dichloromethane and ethanolic extracts was observed. The aqueous extract remained almost without effects on both enzymes at 2 mg/ml.

### Anthelmintic Activity

Among human helminth infections, schistosomiasis is the most prevalent with regard to mortality and the third most harmful tropical disease in the world. An ethanol leaf extract of *E. griseum* was tested *in vitro* against trematodes (*Echinostoma caproni* and *Schistosoma mansoni*) and nematodes (*Ancylostom aceylanicum, Heligmosomoides bakeri* and *Trichuris muris*). The extract reduced the motility of third-stage larvae (L3) of *H. bakeri* by at least 80% at a minimal lethal concentration (MLC) of 20 μg/ml 48 h post-incubation, caused death of newly transformed *S. mansoni* schistosomula and adults with MLC values of 20 and 40 μg/ml, respectively, exhibited a moderate activity against L3 of *A. ceylanicum* 48 h post-incubation and no activity on adult *E. caproni* and *T. muris* (MLC: 2 mg/ml). *In vivo*, mice harboring adult *S. mansoni, E. caproni* and *T. muris* were used. An oral single dose of the extract (400 mg/kg) 49 days post-infection displayed a moderate activity in chronic S. *mansoni* infection (reduction of total worm burden 49.5% and of female worm burden 48.9%), whereas no activity was observed against *E. caproni* and *T. muris* at 400 and 800 mg/kg, respectively (Koné et al., 2012).

## Toxicological Evaluation

Although medicinal plants are widely used and assumed to be safe, however, they can potentially be toxic (Nasri and Shirzad, 2013; Awounfack et al., 2016). “*If herbs have an effect, they are also likely to have a side effect*” (Lanini et al., 2012). In line with this, the safety evaluation of medicinal plants—even used since centuries—needs to be performed. *In vitro* and *in vivo* studies have been carried out aiming at the evaluation of the safety properties of some *Eriosema* species.

In the Ames test on *Salmonella typhimurium* strain TA98 with and without S9 metabolic activation, a water extract of the roots of *E. cordatum* at concentrations up to 5,000 μg/ml was non-mutagenic as shown by the average His^+^ revertant colonies (Ndhlala et al., 2011).

According to Koné et al. (2012), an ethanolic leaf extract of *E. griseum* displayed no toxicity against L6 rat skeletal myoblast cells. Awouafack et al. (2013a), evaluated the cytotoxicity of an ethanolic twig extract of *E. robustum* and compounds thereof against normal monkey Vero cells. In the MTT assay, the extract showed a low cytotoxicity (IC_50_ of 53.45 μg/ml). Among isolates obtained from this extract, **88** and **107** were not cytotoxic, **17**, **41**, **87** and **101** displayed a low cytotoxicity (21.87 ≤ IC_50_ ≤ 91.52 μg/ml), while **16** was moderately cytotoxic (IC_50_ of 13.20 μg/ml).

*In vivo*, the acute oral toxicity of an ethanol root extract (EEC) of *E. chinense* and its chloroform fraction (CEC) was evaluated in female rats following the OECD (Organization for Economic Co-operation and Development) guideline 425. Administration of EEC and its subfractions did not cause any signs of toxicity or mortality up to 2 g/kg during the observation period of 14 days (Prasad et al., 2013b).

In a study by Ateba et al. (2014a), the toxicity of a methanol extract of the aerial parts of *E. laurentii* following guidelines for acute (OECD guideline 423) and subchronic (OECD guideline 407) oral administrations was investigated. A single dose of 2 g/kg of the extract as well as the repetition of the experiment, caused neither toxicological symptoms nor mortality and the LD_50_ was estimated >5 g/kg. In 28-days repeated oral administration, no signs of toxicity were observed phenotypically and in the main organs of rats. The extract only induced a delayed decrease of relative spleen weight and reduced the white blood cell count in males at the highest dose of 400 mg/kg.

Using the Irwin test, Prasad et al. (2017) showed that the acute oral administration of **81** (from the roots of *E. chinense*) up to 300 mg/kg did not result in any sign of behavioral/neurological toxicity or mortality during the observation period. The weight of organs was not affected. Some signs of physical toxicity were observed at the dose of 500 mg/kg.

Nduka et al. (2018) reported LD_50_ values of 3807.9 mg/kg and >5,000 mg/kg for ethanolic and aqueous leaf extracts of *E. psoraleoides*, respectively, following an acute oral treatment.

Toxicological evaluations are of major importance for medicinal plants and herbal products. Without these studies the estimation of their therapeutic potential is limited when the doses to induce non-fatal as well as fatal adverse effects are unknown. Based on the *in vivo* acute toxicity studies, the tested extracts of *E. chinense*, *E. laurentii* and *E. psoraleoides* are placed at category 5 (or unclassified) in the Globally Harmonized Classification System for Chemical Substances and Mixtures (GHS) as adopted by OECD (OECD, 2001). Such products with no deaths up to 2000 mg/kg (estimated LD_50_ > 5000 mg/kg) are considered practically non-toxic (Hodge and Sterner, 1949; Kennedy et al., 1986).

## Conclusion

**FIGURE 4:**
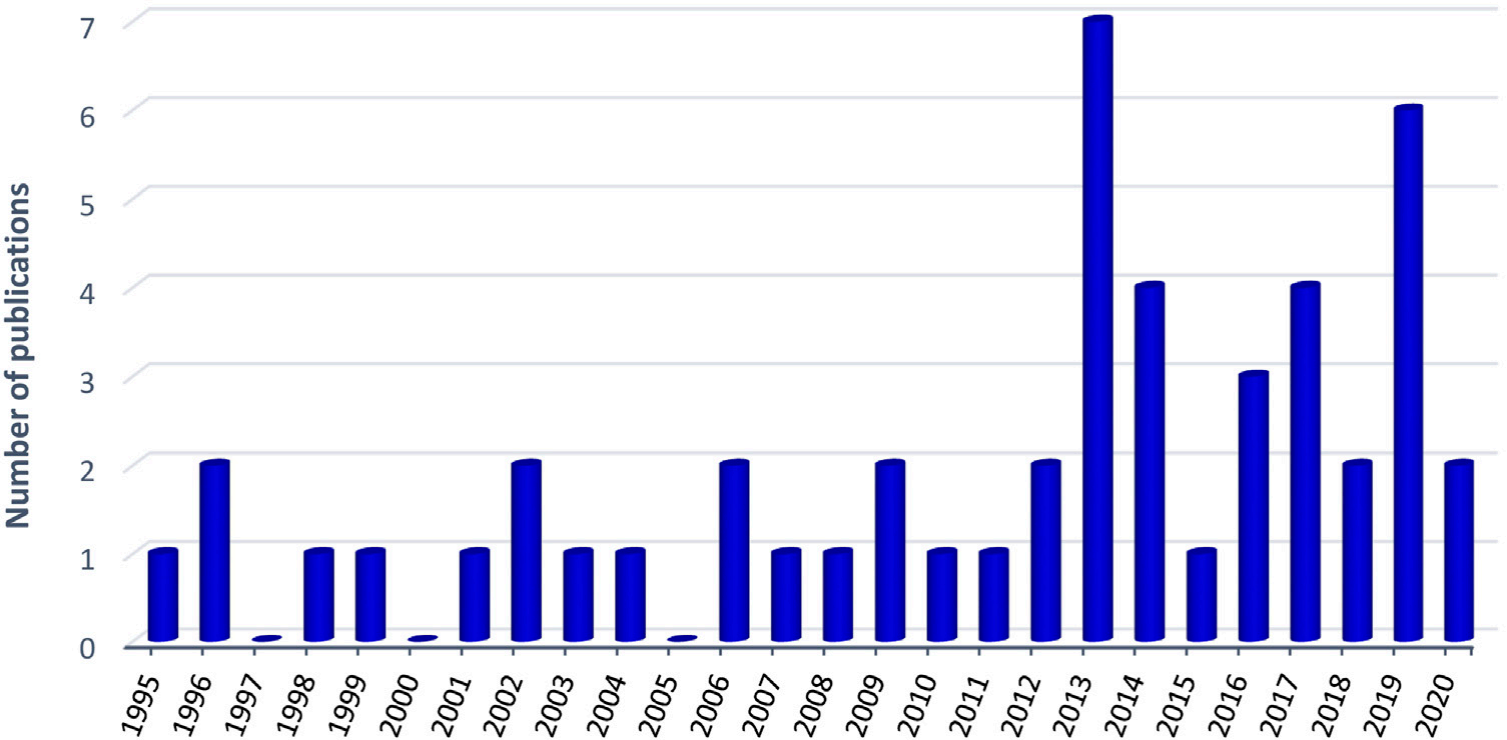
Annual publication rate of *Eriosema* genus since 1995 (phytochemical, pharmacological and toxicological investigation and reviews).

In this paper, we reviewed the available information concerning the traditional uses/ethnopharmacology, phytochemistry, pharmacology and toxicology of the genus *Eriosema*. Twenty-five species (∼16.6% of the genus) recorded in the review are known as traditional medicines and used for nearly 121 ailments. It is evident from anectodical notes that the investigated numbers of species and traditionally treated health problems are so far lower than the reality. Moreover, around 75% of the traditional indications of these plants have not been studied yet in respective pre-clinical studies, indicating that more scientific investigations are necessary. Apart from data on ethnopharmacology, only 49 papers dealing with the phytochemical, pharmacological and toxicological investigation and reviews on *Eriosema* species and compounds occurring in this genus have been published in 25 years (Figure 4). The mean rate of approximately 2 publications per year is very low. Despite the high reputation of several species for curing or alleviating ED/impotence, especially in Africa, the investigation is only limited to two papers on *E. kraussianum* which have been published more than 15 years ago indicating an underexplored aspect that requires more research attention. Concerning phytochemistry, the paper covers a total of 107 compounds mainly belonging to the classes of flavonoids, chromones and terpenoids. The pharmacological activities of extracts and pure compounds from this genus focused on the management of erectile dysfunction, pre-eclamptic complications and anti-diabetic, estrogenic, hypolipidemic, anti-oxidant, anti-microbial, anti-osteoporosis, anthelmintic, anti-diarrheal and anti-cancer effects as well as cyclooxygenase and acetylcholinesterase inhibitory properties. However, there is lack of studies dealing with the in-depth mechanisms involved in the observed activities *in vitro* and in animals. Moreover, no clinical study has been carried out till now. Beyond the pharmacological activities, the toxicity evaluation of medicinal plants is of high importance for their valorization. *In vivo*, acute toxicity carried out with *E. chinense*, *E. laurentii* and *E. psoraleoides* indicated a low toxicity of investigated extracts.
